# Barriers to the prevention of violence against children identified by healthcare professionals: a scoping review

**DOI:** 10.1590/1980-220X-REEUSP-2025-0375en

**Published:** 2026-06-01

**Authors:** Joana Rita Guarda da Venda Rodrigues, Sílvia Vinagre Luz, Susana Margarida Rodrigues Custódio, Ana Isabel dos Santos Pastorinho, Susana Queiroz Santos, Ana Sofia Lopes Caldeira, Juan Diego Ramos-Pichardo, Maria do Céu Barbieri-Figueiredo

**Affiliations:** 1 Centro de Investigação Inovação e Desenvolvimento em Enfermagem de Lisboa (CIDNUR), Escola Superior de Enfermagem, Universidade de Lisboa, Lisboa, Portugal.; 2 Escola Superior de Enfermagem da Universidade de Lisboa, Lisboa, Portugal.; 3 Hospital Dona Estefânia, Unidade Local de Saúde de São José, Cirurgia Pediátrica e Unidade de Queimados, Lisboa, Portugal.; 4 Escola Superior de Saúde, Politécnico de Leiria, Leiria, Portugal.; 5 Centro de Inovação em Tecnologias e Cuidados da Saúde (ciTechCare), Politécnico de Leiria, Leiria, Portugal.; 6 Hospital Dona Estefânia, Unidade Local de Saúde de São José, Serviço de Urgência Pediátrica Polivalente, Lisboa, Portugal.; 7 Hospital de São Francisco Xavier, Unidade Local de Saúde Lisboa Ocidental, Pediatria, Lisboa, Portugal.; 8 Universidad de Huelva, Faculdad de Enfermería, Huelva, España.; 9 Grupo de Investigación HIGIA (CTS-500), Universidad de Huelva, España.; 10 Universidade do Porto, Escola Superior de Enfermagem, RISE-Health, Porto, Portugal.

**Keywords:** Child Abuse, Violence, Health Personnel, Family, Review, Maus-Tratos Infantis, Violência, Pessoal de Saúde, Família, Revisão

## Abstract

**Objective:**

To map the barriers identified by healthcare professionals to the prevention of violence against children.

**Method:**

This scoping review was conducted in accordance with the JBI methodology for scoping reviews. The search was conducted using the MedLine, CINAHL, Psychology and Behavioral Sciences Collection, ERIC, Cochrane, MedicLatina, Scopus, Web of Science, RCAAP, and MedNar databases.

**Results:**

A total of 1,674 publications were identified, with 45 studies included in the review. Barriers were most frequently reported in relation to the identification and reporting of violence against children. Recurrent challenges included deficits in knowledge and training, professional insecurity, excessive workload, and limited time availability. Cultural, social, and organizational factors also emerged as significant cross-cutting obstacles.

**Conclusion:**

The need for targeted training, improved working conditions, and strengthened inter-institutional collaboration is highlighted as a priority area identified in the literature to address the complex and interconnected barriers faced by healthcare professionals in the prevention and combat of violence against children.

## RESUMO

**Objetivo:**

Mapear as barreiras identificadas por profissionais de saúde para a prevenção da violência contra crianças.

**Método:**

Esta revisão de escopo foi conduzida de acordo com a metodologia JBI para revisões de escopo. A pesquisa foi realizada nas bases de dados MedLine, CINAHL, Psychology and Behavioral Sciences Collection, ERIC, Cochrane, Medicaid Latin, Scopus, Web of Science, RCAAP e MedNar.

**Resultados:**

Identificaram-se 1.674 publicações, das quais 45 estudos foram incluídos na revisão. As barreiras mais frequentemente identificadas estavam relacionadas com a identificação e notificação de situações de violência contra crianças. Os desafios recorrentes incluíram lacunas no conhecimento e na formação, insegurança profissional, sobrecarga de trabalho e disponibilidade limitada de tempo. Fatores culturais, sociais e organizacionais também emergiram como obstáculos transversais significativos.

**Conclusão:**

A necessidade de formação específica, melhores condições de trabalho e reforço da colaboração interinstitucional emerge na literatura como uma área prioritária para ultrapassar as barreiras complexas e interrelacionadas que os profissionais de saúde vivenciam na prevenção e no combate à violência contra crianças.

## INTRODUCTION

Violence against children encompasses all forms of violence inflicted on individuals under the age of 18^(1)^. Approximately 400 million young children worldwide - equivalent to six in ten children under the age of 5 - regularly suffer physical punishment and/or psychological violence at the hands of parents and caregivers^(2,3)^.

According to the Convention on the Rights of the Child, the use of violence against children constitutes a violation of their rights^(4)^. Recognizing the severity of this issue, as part of the 2030 Agenda for Sustainable Development, Target 16.2 aims to eliminate abuse, exploitation, trafficking, and all forms of violence and torture directed at children^(5)^.

A large proportion of violence against children involves various forms of interpersonal violence, which tend to occur at different stages of child development. Child maltreatment is a type of violence against children that involves abuse and neglect. It includes “all types of physical and/or emotional ill- treatment, sexual abuse, neglect, negligence and commercial or other exploitation, which results in actual or potential harm to the child’s health, survival, development or dignity in the context of a relationship of responsibility, trust or power”^(3)^. Evidence from various geographical and cultural contexts shows that violence against children is preventable^(1)^.

Experiencing violence during childhood has profound and long-lasting consequences for health and well-being, negatively affecting physical, emotional, social, and psychological development throughout life^(1,6,7)^. Violence impairs a child or adolescent’s ability to understand, express, and recognize their emotions, potentially leading to social isolation and, consequently, an increased risk of addictive and high-risk behaviors, depression, anxiety, and suicidal ideation^(8,9)^.

There is numerous risk factors associated with child maltreatment, such as the child’s age, the presence of health and/or behavioral problems, unrealistic parental expectations, unplanned pregnancies, and a family history of violence^(10)^. Conversely, protective factors have also been identified, such as the practice of positive parenting, the presence of a secure attachment between the child and their family, an effective family and social support network, and easy access to community services, including social, educational, and health services^(10,11,12)^. In 2016, recognizing the devastating impact of violence, the United Nations Secretary-General launched the Global Partnership to End Violence Against Children^(11)^. Thus, due to their close contact with both the child and the family, healthcare professionals play a central role in child maltreatment prevention, intervention, and reporting^(1,11)^. Key disciplines include nurses, midwives, social workers, psychologists, occupational therapy, physiotherapy, speech pathology, and medical professionals - as general practitioner, pediatrician, and psychiatrist^(13)^.

These professionals’ intervention has been shown to impact children’s development and well-being^(14)^, reinforcing the importance of ensuring they are adequately prepared to advocate for the rights and protection of children and adolescents^(1,15)^. However, evidence indicates that despite its high prevalence, violence against children often remains hidden, invisible, or underreported^(9,10)^, which may compromise the effectiveness of interventions in this complex phenomenon^(9)^. Studies show that healthcare professionals often feel unprepared to address such cases due to a lack of appropriate knowledge and skills^(16,17)^. Several studies emphasize the importance of training providers in matters of child abuse and violence against children^(15)^. Although some barriers affecting healthcare professionals’ ability to prevent, identify, intervene in, and report cases of violence against children have already been identified, they are scattered throughout the literature. Across different global contexts, these barriers extend beyond individual knowledge gaps and include high workloads, limited time availability, unclear institutional and legal procedures, insufficient organizational support, cultural norms, and fragmented intersectoral coordination^(15,16,17)^. These challenges may constrain healthcare professionals’ capacity to prevent violence against children in their day-to-day practices, despite the recognized relevance of their intervention in child protection.

A preliminary search was conducted in the MedLine, Cochrane Database of Systematic Reviews, JBI Evidence Synthesis, and Open Science Framework databases, and no current or ongoing systematic reviews or scoping reviews were identified on the topic, specifically regarding the identification of barriers to the prevention and response to violence against children by healthcare professionals. Although previous reviews on related topics exist, their scope remains limited in terms of professional, conceptual, or geographical coverage. Solem et al.^(18)^ focused exclusively on the barriers faced by social workers in England in identifying and responding to cases of child neglect, while Wilson and Lee^(19)^ analyzed factors associated with mandatory reporting behaviors. Owaidah et al.^(9)^, in turn, explored the factors influencing child maltreatment underreporting in Saudi Arabia. In this context, the present scoping review distinguishes itself by aiming to comprehensively and interprofessionally map the barriers identified by healthcare professionals in the prevention and response to violence against children, with particular emphasis on the prevention, identification, and reporting dimensions. By providing a comprehensive and up-to-date overview of the international evidence, this review may help identify gaps in existing literature, support a more informed and integrated response to the phenomenon of violence against children, and guide future strategies in training, professional practice, and research. Accordingly, it seeks to answer the following review question: what are the barriers to preventing violence against children as identified by healthcare professionals? Additionally, it aims to address the sub-question: what are the barriers to the identification, reporting, and intervention in situations of violence against children as identified by healthcare professionals? In line with these aims, this review aims to map the barriers identified by healthcare professionals in the prevention of violence against children.

## METHOD

This scoping review was conducted following the methodology recommended by the JBI^(20)^ and in accordance with the Preferred Reporting Items for Systematic Reviews and Meta-Analyses extension for Scoping Reviews (PRISMA-ScR) checklist^(21,22)^ associated with the PRISMA 2020 flowchart^(23)^. The review protocol was written and analyzed by the authors, and registered with the Open Science Framework (https://osf.io/8nwx7/).

### Eligibility Criteria

The eligibility criteria for the studies were defined based on the PCC mnemonic (Population, Concept, and Context). Studies were included if the population consisted of healthcare professionals, such as nurses, midwives, social workers, psychologists, occupational therapy, physiotherapy, speech pathology, and medical professionals - as general practitioner, pediatrician, and psychiatrist. Regarding the concept, this review examines studies that address barriers to prevent and combat situations of violence against children as identified by healthcare professionals. All healthcare settings were considered, without any limitation. This review included qualitative, quantitative, or mixed type studies as well as literature reviews and gray literature. Documents in all languages, with no restrictions on publication date, were considered. Studies that did not explicitly focus on violence against children were excluded.

### Information Sources

To identify the studies, the Medical Literature Analysis and Retrieval System Online (MedLine Ultimate) (via EBSCO), Cumulative Index of Nursing and Allied Health Literature (CINAHL Ultimate) (via EBSCO), Psychology and Behavioral Sciences Collection, ERIC, Cochrane Central Register of Controlled Trials, MedicLatina, Scopus and Web of Science electronic databases were used. To identify unpublished studies/gray literature, a search was conducted in *Repositório Científico de Acesso Aberto de Portugal* (RCAAP) and MedNar.

The search process encompassed three stages. In the first stage, searches were conducted on the EBSCOhost WEB platform, and the MedLine Ultimate and CINAHL Ultimate electronic databases were used. Natural language search terms were used to identify the keywords used in titles and abstracts, as well as indexing terms. In the second stage, the natural words, keywords, and indexing terms listed were combined with Boolean operators and, when possible, an asterisk operator (*), to form the search expression, which was adapted to the specificities of each database data or repository. The combined terms used included: barrier* OR difficult* OR “child abuse prevention” OR “child abuse intervention” OR “child abuse control” AND “Health Personnel+” OR “Pediatric Nurse Practitioners+” AND “violence against child*” OR “Child Abuse” OR “child maltreatment” OR mistreatment OR violence (Chart 1). The search was conducted between July and October 2024. In the third stage, bibliographic references in the previously identified records were analyzed. Unpublished studies and gray literature were also searched in library repositories.

**Chart 1:** Scoping review search strategies – Lisbon, Portugal, 2024.

Database | search date	Search strategy	Results
CINAHL Ultimate (via EBSCO) July, 2024	(TI ( barrier* OR difficult* OR child abuse prevention OR child abuse intervention” OR child abuse control) OR AB (barrier* OR difficult* OR child abuse prevention” OR child abuse intervention OR child abuse control”)) AND (MH “Health Personnel+”) OR (MH “Pediatric Nurse Practitioners+”) AND (TI ( child maltreatment OR mistreatment OR violence against child*) OR AB (child maltreatment OR mistreatment OR violence against child*) OR (MH “Child Abuse”) OR (MH “Violence”))	284
MedLine Ultimate (via EBSCO) July, 2024	((((((“barrier*” [Title/Abstract]) OR “difficult*” [Title/Abstract]) OR “child abuse prevention” [Title/Abstract]) OR “child abuse intervention” [Title/Abstract]) OR “child abuse control” [Title/Abstract]) AND (((“Health Personnel+”[Mesh]) OR “Pediatric Nursing+”[Mesh]) AND ((((((“child maltreatment” [Title/Abstract]) OR “mistreatment” [Title/Abstract]) OR “violence against child*”[Title/Abstract]) OR (“Child Abuse”[Mesh])) OR (“Violence”[Mesh]))	355
Scopus October, 2024	(TITLE-ABS-KEY ( barrier* ) OR TITLE-ABS-KEY ( difficult* ) OR TITLE-ABS-KEY ( “child abuse prevention” ) OR TITLE-ABS-KEY ( “child abuse intervention” ) OR TITLE-ABS-KEY ( “child abuse control” ) AND TITLE-ABS-KEY ( “Health Personnel” ) OR TITLE-ABS-KEY ( “Pediatric Nurse Practitioners” ) AND TITLE-ABS-KEY ( “child maltreatment” ) OR TITLE-ABS-KEY ( mistreatment ) OR TITLE-ABS-KEY ( “violence against child*” ) OR TITLE-ABS-KEY ( “Child Abuse” ) OR TITLE-ABS-KEY ( violence ) )	649
Psychology and Behavioral Sciences Collection July, 2024	(TI ( barrier* OR difficult* OR “child abuse prevention” OR “child abuse intervention” OR “child abuse control” ) OR AB ( barrier* OR difficult* OR “child abuse prevention” OR “child abuse intervention” OR “child abuse control” ) ) AND (TI ( “Health Personnel” OR “Pediatric Nurse Practitioners” ) OR AB ( “Health Personnel” OR “Pediatric Nurse Practitioners” ) ) AND ( TI ( “child maltreatment” OR mistreatment OR “violence against child*” OR “Child Abuse” OR violence ) OR AB ( “child maltreatment” OR mistreatment OR “violence against child*” OR “Child Abuse” OR violence ) )	0
Cochrane Central Register of Controlled Trials July, 2024	(TI ( barrier* OR difficult* OR “child abuse prevention” OR “child abuse intervention” OR “child abuse control” ) OR AB ( barrier* OR difficult* OR “child abuse prevention” OR “child abuse intervention” OR “child abuse control” ) ) AND (TI ( “Health Personnel” OR “Pediatric Nurse Practitioners” ) OR AB ( “Health Personnel” OR “Pediatric Nurse Practitioners” ) ) AND ( TI ( “child maltreatment” OR mistreatment OR “violence against child*” OR “Child Abuse” OR violence ) OR AB ( “child maltreatment” OR mistreatment OR “violence against child*” OR “Child Abuse” OR violence ) )	0
MedicLatina July, 2024	(TI ( barrier* OR difficult* OR “child abuse prevention” OR “child abuse intervention” OR “child abuse control” ) OR AB ( barrier* OR difficult* OR “child abuse prevention” OR “child abuse intervention” OR “child abuse control” ) ) AND (TI ( “Health Personnel” OR “Pediatric Nurse Practitioners” ) OR AB ( “Health Personnel” OR “Pediatric Nurse Practitioners” ) ) AND ( TI ( “child maltreatment” OR mistreatment OR “violence against child*” OR “Child Abuse” OR violence ) OR AB ( “child maltreatment” OR mistreatment OR “violence against child*” OR “Child Abuse” OR violence ) )	0
Web of Science October, 2024	“child maltreatment” AND “health personnel”	7
RCAAP October, 2024	“child maltreatment”	65
CAPES October, 2024	TI (“child maltreatment” AND “health personnel”)	4
MedNar October, 2024	TI (“child maltreatment”) Limit to Available Full-Text	325

### Data Extraction

Search results were exported to the Mendeley Desktop reference manager (version 1.19.8), where duplicate records were identified and removed. Subsequently, to support the selection process, the records were imported into the Qatar Computing Research Institute platform (Rayyan QCRI), which was used to organize the records and facilitate blinded screening. Eligibility was assessed by two independent reviewers who screened the titles and abstracts against the predefined inclusion and exclusion criteria. Eligibility was assessed by two independent reviewers who screened the titles and abstracts against the predefined inclusion and exclusion criteria, with disagreements resolved through discussion or consultation with a third reviewer. Articles that met the eligibility criteria were retrieved in full, and the full text was independently assessed in detail for eligibility by two or more reviewers.

### Results Summary

Data synthesis from records that met the inclusion and exclusion criteria was conducted independently by two authors, using data extraction instruments developed by the research team, in alignment with the objectives and review question of this scoping review. The extracted information was entered into a Microsoft Excel spreadsheet. The results are presented in summary charts.

After assessing each other’s records, the authors highlighted the aspects that needed clarification. These concerns were addressed through discussion, and the results were presented.

## RESULTS

**Figure 1:**
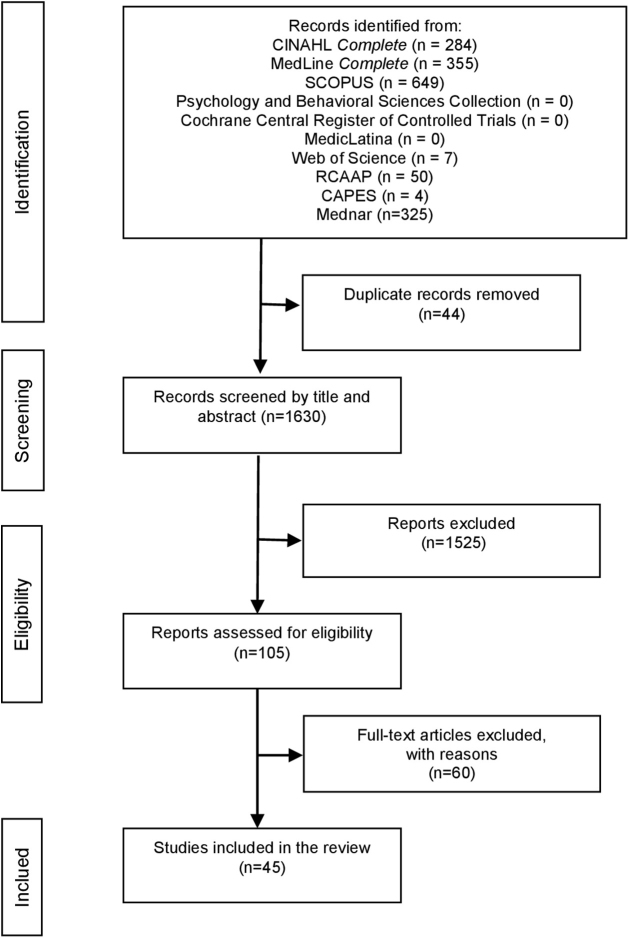
Flowchart for the search and study selection process.

A total of 1,674 articles were identified across the databases, of which 44 were removed as duplicates. Following title and abstract screening, 105 articles were selected for full-text assessment. The PRISMA-ScR methodology^(23)^ (Figure 1) was used to systematize the study inclusion process. Charts 2, 3, 4, 5, and 6 present a summary of the main findings. The records excluded during title and abstract screening did not meet the eligibility criteria established for this review, as they did not focus on violence against children, did not involve healthcare professionals as defined, or did not examine barriers in the prevention and combat of violence against children as identified by healthcare professionals. In all charts, the study numbers correspond to the references as numbered in the Vancouver-style reference list.

**Chart 2:** General characteristics of reviewed studies – Lisbon, Portugal, 2025.

Study (year)	Country	Study design	Objective(s)	Setting	Type of violence against children
Antwi et al. (2019)^(24)^	Ghana	Qualitative	Explore external influences on Ghanaian radiologist decision-making to report suspected child physical abuse; identify cultural influences and beliefs regarding reporting of child physical abuse by radiologists in Ghana.	Hospital	Physical abuse
Antwi et al. (2021)^(25)^	Ghana	Qualitative	Explore the internal factors that lead to bystander attitudes toward reporting suspected physical abuse among Ghanaian radiologists.	Hospital	Physical abuse
Beck and Ogloff (1995)^(26)^	Canada	Mixed method	Investigate specific factors related to child abuse reporting, controlling for some of the limitations of previous research.	Faculty of Psychology directory	Physical abuse Emotional abuse Sexual abuse Neglect
Beck et al. (2015)^(27)^	United States of America	Mixed method	Assess knowledge gaps and training needs of medical providers; demonstrate the importance of provider training to meet the pediatric sex trafficking victim’ specific needs; and highlight barriers to identifying and responding to victims.	Hospital	Sex trafficking
Berchtold et al. (2023)^(28)^	Switzerland	Mixed method	Examine the obstacles and enablers for detecting and reporting child abuse among nursing and medical staff in pediatric emergency departments and pediatric surgery departments.	Hospital	Child abuse
Carson (2018)^(29)^	United States of America	Quality improvement project	Implement an evidence-based screening program that includes provider education about child physical abuse, a systematic screening protocol, and use of the validated Escape Instrument.	Pediatric Emergency Department	Physical abuse
Crichton et al. (2016)^(30)^	Colombia	Qualitative	Assess the use and characteristics of screening protocols for physical abuse in children’s hospitals and determine attitudes toward the use of such tools.	Pediatric hospital	Physical abuse
Egry et al. (2017)^(31)^	Brazil	Qualitative	Understand the perception of healthcare professionals working in primary care on child violence.	Community healthcare services	
Elarousy and Abed (2019)^(32)^	Saudi Arabia	Descriptive	Investigate barriers inhibiting the reporting of suspected cases of child abuse and neglect by nurses in King Abdulaziz Medical City, Jeddah, Saudi Arabia.	Pediatric hospital	Child abuse Neglect
Eniola and Evarts (2017)^(33)^	United States of America	Mixed method	Provide recommendations to improve general practitioners’ confidence in diagnosing child abuse.	American Academy of Family Physicians	
Faryan et al. (2019)^(34)^	Saudi Arabia	Phenomenological	Explore child protection workers’ experiences in Saudi Arabia.		Child abuse Neglect
Fleckman et al. (2021)^(35)^	United States of America	Mixed method	Assess American pediatricians’ preparation, motivation, and barriers related to educating parents about corporal punishment and effective child discipline.	United States National Database	Physical and emotional abuse
Galindo et al. (2017)^(36)^	Brazil	Qualitative	Analyze nurses’ knowledge about the types of child and adolescent violence, identifying the conduct, difficulties and possible prevention and health promotion actions that are designed in Family Health Strategies.	Community healthcare services	Sexual abuse Physical violence Neglect
Gunn et al. (2005)^(37)^	United States of America	Mixed method	Identify factors associated with pediatricians’ decision not to report suspected child abuse.	Department of Health	Child abuse Neglect
İnanici et al. (2020)^(38)^	Turkey	Mixed method	Investigate physicians’ experiences with abuse cases, their assessments of abuse risk factors, and their attitudes toward becoming more trained on the subject.	Community healthcare services	Child abuse Neglect
Kent et al. (2011)^(39)^	Ireland	Qualitative	Explore a group of public health nurses’ views on their role with preschool children.	Community healthcare services	
Khanjari et al. (2021)^(40)^	Iran	Quasi-experimental	Determine the effect of an educational program on Iranian nurses’ recognition and intention to report cases of child abuse.	Pediatric emergency department and pediatric ward	
Konijnendijk et al. (2014)^(41)^	Netherlands	Qualitative	Identify factors related to the characteristics of the guidelines, the user, the organization, and the sociopolitical context that facilitate or hinder adherence to child abuse prevention guidelines.	Children’s Health Organization	Child abuse
Land and Barclay (2008)^(42)^	Australia	Qualitative	Explore, with a small sample of child health, pediatric and school nurses, their understanding and knowledge of child abuse and neglect; identify these nurses’ perceptions about their role and involvement in protecting children; and identify any perceived barriers to nurses playing a role in protecting children.	Community healthcare services	Child abuse **Neglect**
Leung et al. (2011)^(43)^	Hong Kong	Cross-sectional	Investigate Hong Kong general practitioners’ attitudes and behaviors towards reporting child abuse and their views on the introduction of a mandatory reporting system.		Child abuse
Lines et al. (2020)^(44)^	Australia	Qualitative	Report nurses’ perceptions of how organizational systems and hierarchies shaped their ability to respond to child abuse and neglect.	Community healthcare services and hospital	Child abuse Neglect
Lines et al. (2024)^(45)^	Australia	Qualitative	Build a broader and more comprehensive understanding of the barriers faced by nurses and midwives when safeguarding children.	Child-focused settings	
Lobato et al. (2012)^(46)^	Brazil	Case study	Analyze family health team professionals’ perception about violence in their area of activity and the challenges in reporting cases and providing care to families in situations of violence.	Community healthcare services	Domestic violence against children and adolescents Physical abuse Neglect Abandonment
Lynne et al. (2015)^(47)^	United States of America	Mixed method	Understand why emergency medical services providers may stop reporting suspected abuse despite mandatory reporting policies.	General emergency department	
Mandadi et al. (2021)^(48)^	United States of America	Cross-sectional	Describe pediatric emergency medicine physicians’ knowledge, training, confidence, and barriers in recognizing and reporting suspected child abuse.	Pediatric emergency	Child abuse Neglect
Mostovoy et al. (2024)^(49)^	Israel	Quantitative	Explore ophthalmologists’ attitudes, knowledge, and willingness to report child abuse.	Ophthalmologists’ Association	Child abuse
Nouman et al. (2020)^(50)^	Israel	Qualitative	Investigate the different ways in which healthcare professionals working in community services manage compulsory reporting, their basis and experience with formal demands.	Largest healthcare management organization	Child abuse Neglect
Owaidah et al. (2022)^(9)^	Saudi Arabia	Review	Determine different factors that influence child abuse underreporting in Saudi Arabia.		Child abuse Neglect
Paavilainen et al. (2002)^(51)^	Finland	Mixed method	Establish whether pediatric nurses and physicians at a teaching hospital need additional training in identifying child physical abuse and whether there is a need to develop health education on child abuse issues.	University hospital	Child abuse Physical abuse Psychological abuse Sexual abuse Neglect
Paavilainen et al. (2002)^(52)^	Finland	Quantitative	Observe how nurses and physicians at a university hospital assessed their ability to identify child abuse while caring for these children.	University hospital	
Patrick et al. (2020)^(53)^	United Kingdom	Audit	Identify and address barriers to reporting (child) protection concerns among hospital staff.	Hospital	Child abuse Neglect
Regnaut et al. (2015)^(54)^	France	Mixed method	Assess levels of knowledge of risk factors for child abuse by family physicians and the attention physicians pay to these risk factors.	Private clinic	Child abuse
Riese et al. (2014)^(55)^	United States of America	Epidemiological	Examine resident physicians’ attitudes, behaviors, and barriers toward preventing youth violence while caring for violently injured youth in the acute care setting.	Pediatric hospital	Youth violence
Rowse (2009)^(56)^	England	Qualitative	Explore the experiences of nurses from a hospital pediatric ward, who were directly involved in child protection cases, to understand their support needs and suggest developments in training and support.	Pediatric hospital	
Roy et al. (2022)^(57)^	United Kingdom	Mixed method	Assess the feasibility and acceptability of the IRIS+ model for children.	Hospital	Domestic violence and abuse
Russell et al. (2004)^(58)^	Northern Ireland	Mixed method	Assess primary healthcare professionals’ perception and ability to recognize child physical abuse during practice.	Community healthcare services	Physical abuse
Soh et al. (2023)^(59)^	Australia	Mixed method	Identify barriers to recognizing and reporting potential child abuse by medical officers and nursing staff.	Hospital	Physical abuse Neglect Exposure to family violence Sexual abuse Emotional abuse
Solem et al. (2020)^(18)^	England	Serious case reviews	Explore the barriers that exist for social workers in England to identify and respond to neglect in a timely, appropriate and effective manner.	National Case Review Repository	Neglect
Tiyyagura et al. (2015)^(60)^	United States of America	Qualitative	Explore general emergency department providers’ experiences with screening and reporting child abuse and neglect; identify barriers and facilitators to detecting child abuse and neglect in the emergency setting.	General emergency department	Child abuse Neglect
Tiyyagura et al. (2017)^(61)^	United States of America	Qualitative	Explore barriers and facilitators to recognizing and reporting child abuse and neglect by pre-hospital care providers.	Pre-hospital	Child abuse Neglect
Tiyyagura et al. (2019)^(62)^	United States of America	Qualitative	Understand the program’s strengths and challenges; explore the factors that influenced its implementation.	Academic Medical Center	Child abuse Neglect
Vilensky et al. (2022)^(63)^	Israel	Qualitative	Identify the barriers that prevent community nurses in Israel from reporting cases of child maltreatment.	Health management services primary care clinics	Child abuse
Wißmann et al. (2019)^(64)^	Germany	Mixed method	Examine pediatricians’ reporting behavior in cases of child abuse and neglect and their attitudes toward mandatory reporting.	Private clinic	Child abuse Neglect
Willis and Horner (1987)^(65)^	United States of America	Mixed method	Estimate the level of suspicion and reporting of child sexual abuse by family physicians; identify possible cognitive, behavioral, and experiential barriers to suspecting and reporting of child sexual abuse.	East Carolina University School of Medicine Department of Family Medicine	Sexual abuse
Wilson and Lee (2021)^(19)^	United States of America	Review	Review existing literature focusing on factors that relate to child care professionals’ mandatory reporting behaviors of child abuse and neglect, with specific attention to nurses; summarize study findings; provide implications for clinical practice.		Child abuse Neglect

**Chart 3:** Barriers to preventing violence against children – Lisbon, Portugal, 2025.

Study	Barriers to preventing violence against children
Egry et al.^(31)^	- Reduced number of professionals.
Fleckman et al.^(35)^	- Lack of training, knowledge, and confidence in addressing child discipline; - Cultural sensitivity concerns, perceived low priority of the topic, discomfort in discussing the issue, and doubts regarding the effectiveness of the intervention; - Limited time availability, absence of financial reimbursement, and insufficient institutional support.
Kent et al.^(39)^	- Challenges in risk definition and high case volume.
Konijnendijk et al.^(41)^	- Poor familiarity with guidelines and low self-efficacy in ambiguous cases, caregiver communication, follow-up planning, and interagency information sharing; - Caregiver non-cooperation and greater uncertainty when discussing suspected abuse in home settings; - Flaws in electronic medical records and poor interagency cooperation.
Lines et al.^(45)^	- Need for professional development and access to clinical supervision; - Psychosocial influences (burnout, compassion fatigue, personal factors), exclusion from safeguarding discussions, and mismatch between standardized practices and family needs; - High workloads, unrealistic expectations, understaffing, structural and communication gaps, absence of interdepartmental agreements, and limited service availability.

**Chart 4:** Barriers to identifying violence against children – Lisbon, Portugal, 2025.

Study	Barriers to identifying violence against children
Antwi et al.^(25)^	- Diffusion of responsibility.
Beck et al.^(27)^	- Lack of training and awareness on sex trafficking; - Subject sensitivity and victims’ fear of reporting; - Absence of policy/guidance and insufficient financing/resources.
Berchtold et al.^(28)^	- Lack of experience, knowledge, and confidence in detecting signs and symptoms of child abuse, and difficulty distinguishing accidental from non-accidental events; - Respect for the topic’s taboo nature; - Lack of transparency in the reporting process.
Carson^(29)^	- Limited familiarity of triage staff with the Escape Instrument; - Uncertainty about screening program details and false-positive Escape screens; - Emergency department configuration, time restrictions, delays in child abuse team, consultations, and absence of a dedicated location for documenting screening results.
Crichton et al.^(30)^	- Lack of understanding or awareness of child abuse and difficulty communicating with caregivers in suspected cases; - Perception that screening tools are unnecessary; - Lack of time to develop or complete screening tools, insufficient managerial support, and limited community resources.
Egry et al.^(31)^	- Confusion between neglect and unmet basic needs due to poverty; - Need to strengthen basic healthcare as a reference and support in identifying violence.
Eniola and Evarts^(33)^	- Inexperience, inadequate training, lack of confidence in case assessment and communication with parents; - Language barriers and physicians’ cultural background; - Absence of identification protocols and insufficient time for physical examination.
Galindo et al.^(36)^	- Gaps in nurses’ training and qualifications; - Information obtained indirectly through community members and denial by families of information supporting suspicions; - Overload of duties for nurses.
İnanici et al.^(38)^	- Fear of incorrect identification of abuse, difficulty communicating face to face with the child, problems in controlling emotions, and difficulty managing psychological processes related to the child and/or family; - Feeling alone, fear of being harmed by those involved, and disbelief that the family could mistreat the child; - Workload.
Khanjari et al.^(40)^	- Low level of nurses’ knowledge and limited training in detecting signs and symptoms of child abuse.
Lobato et al.^(46)^	- Concealment of situations by families due to poverty, difficulty identifying negligent attitudes considering the socioeconomic context.
Mandadi et al.^(48)^	- Lack of knowledge, uncertainty in certain clinical presentations, and less training on child neglect than physical abuse; - Availability of social worker, feedback from child protection agencies, and availability of validated screening tool.
Paavilainen et al.^(51)^	- Low awareness of the phenomenon; - Strangeness of the phenomenon; - Intense rhythm of teamwork, lack of jointly agreed guidelines.
Paavilainen et al.^(52)^	- Lack of knowledge and experience; - Sensitivity of the topic, concealment by children and parents, alternative explanations for behaviors, tendency to hide abuse, disbelief that abuse is true, diversity of symptoms; - Work pressure from the team.
Regnaut et al.^(54)^	- Difficulty establishing identification of child abuse.
Riese et al.^(55)^	- Lack of training, not feeling competent; - Lack of time, hospital and extra-hospital resources.
Russell et al.^(58)^	- Inexperience, poor interview techniques; - Fear of incorrect identification, lack of desire to confront the family; - Fear that anonymity would not be maintained.
Soh et al.^(59)^	- Gaps in knowledge and training, not feeling comfortable, and uncertainty about reporting procedures; - Lack of time, lack of support from senior staff, social work, and pediatrics, difficulty finding policy, limited resources and support, and excessive workload.
Tiyyagura et al.^(60)^	- Inability to recognize behavioral indicators of abuse and neglect; - Desire to believe caregivers, personal prejudices, misattribution of behaviors, and lack of ongoing family contact in emergency settings.
Tiyyagura et al.^(61)^	- Discomfort with pediatric clients, uncertainty distinguishing accidental from intentional injuries, and focus on the chief complaint; - Limited opportunity for assessment.
Willis and Horner^(65)^	- Failure to believe abuse occurs at reported frequency and belief that reporting leads to unfavorable consequences; - Lack of confidence in local social services’ ability to manage sexual abuse.

**Chart 5:** Barriers to reporting violence against children – Lisbon, Portugal, 2025.

Study	Barriers to reporting violence against children
Antwi et al.^(24)^	- Fear of physical or spiritual attack, cultural interdependence, and solidarity.
Antwi et al.^(25)^	- Knowledge gaps; - Lack of motivation from senior colleagues, teamwork challenges, and lack of cooperation in suspected abuse cases; - Uncertainty of receiving administrative or legal support from the hospital, workload, and time constraints.
Beck and Ogloff^(26)^	- Lack of sufficient evidence; - Lack of trust in child protection services.
Berchtold et al.^(28)^	- Doubts about identification, feeling unaccountable for reporting, uncertainty of reporting consequences, parental protection, medical hierarchy, and interprofessional factors; - Lack of time, forgetting to report.
Egry et al.^(31)^	- Lack of knowledge regarding referral processes and deficit of knowledge about violence; - Need for clearer referral guidelines, insufficient healthcare network, lack of organization, financial resources, and qualified professionals.
Elarousy and Abed^(32)^	- Not knowing how to report, belief in being able to intervene more effectively than specialized teams; - Uncertainty of abuse, lack of corroboration from others, negative experiences after reporting, perception that injury was minor, and absence of legal consequences for non-reporting.
Gunn et al.^(37)^	- Lack of knowledge about whistleblowing laws; - Concern about consequences for the provider or child, fear of being wrong, uncertainty of maltreatment occurrence, previous bad experiences with child services agencies; - Time and effort involved in reporting.
İnanici et al.^(38)^	- Lack of knowledge of forensic reporting rules, difficulty preparing the report, and lack of knowledge on notification procedures.
Kent et al.^(39)^	- Difficult relationships and frequent changes of social workers, and lack of adequate response after referrals.
Khanjari et al.^(40)^	- Ethical, social, cultural, and religious restrictions, including concepts of honor, modesty, and shame.
Leung et al.^(43)^	- Lack of sufficient evidence, reluctance to engage with legal systems, potential harm to child or family from reporting, and concern over anonymity; - Reporting process is time-consuming.
Lynne et al.^(47)^	- Not knowing what should be reported or how to report and uncertainty about anonymity; - Belief others will report, fear of causing harm, previous negative experiences, and belief in drastic social service measures; - Lack of time, delays in reporting, and medical emergencies taking priority.
Mandadi et al.^(48)^	- Inadequate knowledge of reporting laws and deadlines; - Insufficient time for reporting, lack of social worker availability, lack of feedback or action from child protection services.
Mostovoy et al.^(49)^	- Lack of skills or training; - Concern for child’s feelings, belief children will not cooperate, cultural or language barriers, concerns for personal safety, belief reporting will not help, and negative professional and personal experiences; - Lack of time and avoidance of legal involvements.
Nouman et al.^(50)^	- Lack of identification testing and insufficient feedback from child protection services.
Owaidah et al.^(9)^	- Inability to identify evidence or indicators, lack of knowledge due to insufficient training, belief minor injuries do not warrant reporting, and lack of confidence among inexperienced professionals; - Stigma, cultural norms, misunderstanding of laws, lack of awareness of consequences, uncertainty about family response, fear of consequences, previous negative experiences, and fear of further harm to the child; - Lack of policy structure, ineffective training implementation, and absence of legal implications for non-reporting.
Patrick et al.^(53)^	- Concerns for child and personal safety, uncertainty in identification, confidentiality, and legal ramifications.
Regnaut et al.^(54)^	- Lack of awareness of professional resources; - Fear of incorrect identification, difficulty controlling emotions, feeling alone, fear of threats, and family socioeconomic factors; - Time needed to complete reports, difficulty preparing reports, and procedural barriers for rape reporting.
Riese et al.^(55)^	- Lack of training; - Lack of time, hospital, and extra-hospital resources.
Rowse^(56)^	- Lack of response or positive action from social workers.
Roy et al.^(57)^	- Lack of time; - Confidentiality concerns.
Russell et al.^(58)^	- Uncertainty in reporting; - Fear of incorrect identification, bureaucracy, hierarchy, lack of sensitivity, and support from social services and colleagues; - Lack of guidelines, workload, and long reporting procedures.
Tiyyagura et al.^(61)^	- Fear of mistakes and of caregiver reactions; - Fast-paced work environment.
Vilensky et al.^(63)^	- Lack of knowledge, training, and legal knowledge; - Uncertainty in identification and reporting consequences, fear of repercussions for patient, family, or staff, and fear of mistaken report.
Wißmann et al.^(64)^	- Lack of experience in identification or handling cases; - Fear of false allegations, uncertainty in identification, belief in singular event, reluctance due to personal ties with parents, fear of legal testimony, safety concerns, cultural or religious injury explanations, and distrust in child protection services’ effectiveness; - Lack of time, insufficient interventions by child protection services, administrative barriers, and belief that own work is more effective.
Willis and Horner^(65)^	- Disbelief in prevalence of abuse and fear of negative consequences from reporting; - Lack of confidence in social services’ ability to manage cases.
Wilson and Lee^(19)^	- Lack of knowledge on how to report cases, deficit in clinical and judgment skills, and lack of awareness; - Cultural norms, rural proximity reducing anonymity, biased professional views, reluctance to report, distrust in agencies, fear of disputes, and emotional challenges; - Deficient reporting infrastructure, lack of centralized system, poor communication, inadequate record-keeping, lack of feedback, absence of explicit guidelines, inadequate assessment tools, insufficient management support, high staff turnover, workload, and lack of reporter protection.

**Chart 6:** Barriers to intervening in violence against children – Lisbon, Portugal, 2025.

Study	Barriers to intervening in violence against children
Beck et al.^(27)^	- Lack of training on sex trafficking and lack of awareness about the phenomenon; - Sensitivity of the subject and victims’ fear of reporting; - Lack of organizational policy/guidance and lack of financing/resources.
Egry et al.^(31)^	- Network performance hampered by sectoral and vertical logic, slow and unbalanced communication and information exchange.
Faryan et al.^(34)^	- Lack of professionals’ knowledge; - Cultural factors; - Lack of resources and organizational capacity, implementation challenges, and inadequate working conditions of frontline staff.
Kent et al.^(39)^	- Concern that involvement in the monitoring and surveillance of families identified as “at risk” could result in a threat to the public health nurses’ friendly image.
Land and Barclay^(42)^	- Subjective decision making and lack of adequate training; - Cultural perspectives, professional protection, personal safety, and professional implications; - Scarce resources, lack of multidisciplinary collaboration, lack of clear protocols, lack of autonomy, and hierarchical structures.
Lines et al.^(44)^	- Fear of making mistakes and inflexible systems; - Poor interconnection of services, duplication, poor coordination, rigid systems, and information sharing hierarchies.
Rowse^(56)^	- Theory-practice gap, lack of knowledge, and support in formal procedures; - Lack of information about case developments.
Solem et al.^(18)^	- Difficulty defining and identifying negligence, insufficient training, and failure to use toolkits due to high staff turnover; - Limited resources, high staff turnover, excessive case numbers affecting child protection plan management, and lack of supervision; - Children’s opinions not heard or considered, professionals succumbing to the “optimism rule”.
Tiyyagura et al.^(62)^	- Variability of institutional support for champions, reliance on individual motivation, role overload for champions, rotation of champions, and variability of front-line provider program knowledge.

The studies included in this review were published between 1987 and 2024, considering the search period conducted between July and October 2024, across 20 different countries: United States of America (13)^(19,27,29,33,35,37,47,48,55,60,61,62,65)^; Saudi Arabia (3)^(9,32,34)^; Australia (4)^(42,44,45,58)^; Brazil (3)^(31,36,46)^; Israel (3)^(49,50,63)^; Ghana (2)^(24,25)^; Finland (2)^(51,52)^; England (2)^(56,59)^; United Kingdom (2)^(53,57)^; Canada (1)^(26)^; Germany (1)^(64)^; Colombia (1)^(30)^; France (1)^(54)^; Netherlands (1)^(41)^; China (1)^(43)^; Northern Ireland (1)^(58)^; Ireland (1)^(39)^; Iran (1)^(40)^; Switzerland (1)^(28)^; and Turkey (1)^(38)^. This translates into considerable sociocultural diversity that, in turn, allows us to adopt a more comprehensive and complete vision.

The included studies were conducted across 20 countries, spanning different continents and sociocultural contexts. More specifically, the studies originated from North America (e.g., United States, Canada), Europe (e.g., United Kingdom, Finland, Netherlands, Germany, France, Switzerland, Ireland), Asia (e.g., Saudi Arabia, Iran, China), the Middle East (e.g., Israel), Africa (e.g., Ghana), and South America (e.g., Brazil). The temporal distribution of studies ranged from 1987 to 2024, with a higher concentration of studies published in the last decade, reflecting increased attention to child protection and professional responsibilities in healthcare settings. This geographical and temporal diversity highlights the breadth of contexts in which barriers to preventing violence against children have been identified.

Concerning study design, 18 qualitative studies were identified^(24,25,30,31,34,36,39,41,42,44,45,46,50,56,60,61,62,63)^, including one case study^(46)^ and one phenomenological study^(34)^. Were also included one quality improvement project^(29)^, one analysis of qualitative responses^(45)^, and one audit^(53)^. A descriptive study was included^(32)^. Additionally, one quantitative study^(52)^, one quantitative correlational survey^(49)^, two cross-sectional studies^(43,48)^, one epidemiological study^(55)^, and one quasi-experimental study^(40)^ were also included. Finally, 15 studies employed mixed methods approaches^(26,27,28,33,35,37,38,47,51,54,59,64,65)^. Three of the included studies are literature reviews^(9,18,19)^.

In relation to the data collection setting, in 20 studies data were collected in hospital settings^(24,25,26,28,29,30,32,40,44,47,48,51,52,53,55,56,57,59,60,62)^. Of these, two refer to university hospitals^(51,52)^; one includes data collected at three community hospitals and one academic medical center^(62)^; and one refers to a large metropolitan teaching hospital, a small metropolitan hospital, and a rural hospital^(59)^. Two studies were conducted in general emergency settings^(47,60)^, four in pediatric hospitals^(30,31,32,55,56)^, three in pediatric emergency departments^(28,29,48)^, and one in both a pediatric emergency department and a pediatric ward^(40)^. Additionally, two studies collected data in private clinics^(54,64)^ and one in a pre-hospital setting^(61)^. One study did not specify the healthcare service where data were collected^(43)^, and another only reported that data were collected from a children’s health organization^(41)^.

In eight studies, data were gathered in community healthcare settings^(31,36,38,39,42,46,58,63)^, and in two studies, data were obtained from both community and hospital settings^(44,45)^. In nine studies, data were collected from individuals working with children or affiliated with healthcare or child protection organizations^(26,33,34,35,37,45,49,50,65)^.

As for the type of violence against children, six studies deal with child abuse in general^(28,41,43,49,54,63)^; one article addresses neglect^(18)^; and 14 delve into child abuse and neglect^(9,19,32,37,38,42,44,48,50,53,60,61,64)^. Five of the studies cover physical abuse^(24,25,29,30,58)^, and one examines physical and emotional abuse^(35)^. With regard to sexual abuse against children, one article examines child sexual abuse^(65)^, and another explores child sex trafficking^(26)^. Five studies address three or more types of violence against children, all of which include physical abuse and neglect, but also including emotional and psychological abuse^(26,51)^, sexual abuse^(26,36,51,59)^, and abandonment^(46)^. One examines domestic violence and abuse^(57)^, and another youth violence^(55)^. Nine of the included articles do not specify the type of abuse^(31,33,34,39,40,45,47,52,56)^.

### Barriers to Preventing Violence Against Children

**Figure 2:**
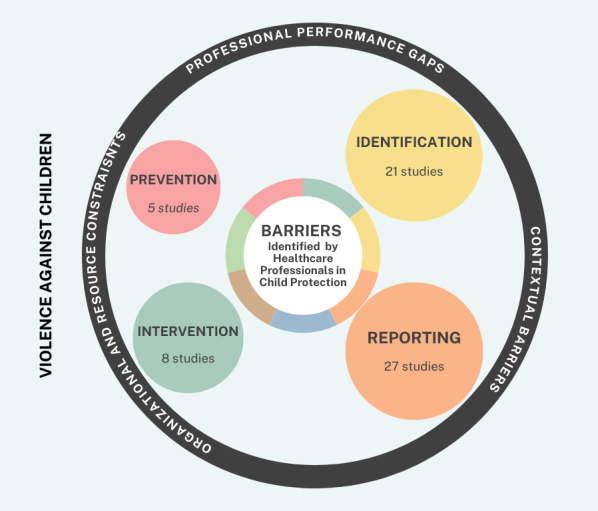
Conceptual mapping of the barriers identified by healthcare professionals in the prevention of violence against children – Lisbon, Portugal, 2025.

This review identified four main types of barriers related to violence against children ((i) barriers to prevention; (ii) barriers to identification; (iii) barriers to reporting; and (iv) barriers to intervention), as detailed in Charts 3, 4, 5, and 6, and synthesized visually in Figure 2.

To enhance analytical clarity, the four types of barriers (prevention, identification, reporting, and intervention) were further synthesized across three transversal dimensions - professional performance gaps, contextual barriers, and organizational and resource constraints -, as illustrated in Figure 2.

## DISCUSSION

This scoping review identified four main types of barriers related to violence against children - prevention, identification, reporting, and intervention -, which reflect different stages of professional action in child protection. Beyond this categorical organization, the synthesis of the findings revealed that these barriers clustered around three transversal dimensions: professional performance gaps; contextual barriers; and organizational and resource constraints. This analytical structure, illustrated in Figure 2, allows for a more integrated understanding of how barriers operate across different phases of prevention and response to violence against children.

The inclusion of studies conducted in 20 countries across multiple continents underscores the substantial sociocultural, legal, and organizational heterogeneity underlying these barriers. Although the specific manifestation of barriers varies according to regional contexts and healthcare system structures, similar patterns emerged, suggesting that many of the challenges faced by healthcare professionals may be cross-cutting. In particular, professional performance gaps and organizational constraints were reported more frequently than contextual barriers, indicating their relative weight in limiting effective prevention, identification, reporting, and intervention practices.

The uneven distribution of studies across barrier types - with reporting and identification barriers being most frequently reported - may reflect greater institutional, legal, and emotional complexity associated with these stages of child protection. This finding is particularly relevant when interpreted in light of Sustainable Development Goal 16.2, which emphasizes the need to strengthen institutional capacity, professional training, and intersectoral coordination to eliminate all forms of violence against children. The persistence of similar barriers across diverse contexts and over time suggests that progress towards this goal remains uneven and that systemic challenges appear to continue to constrain professional action.

Rather than discussing the barriers exclusively according to stages of action, the discussion that follows synthesizes the findings around three transversal dimensions - professional performance gaps, contextual barriers, and organizational and resource constraints -, in order to broaden the discussion and highlight their interconnections.

### Barriers to Preventing Violence Against Children

Barriers to preventing violence against children are identified in five of the included studies^(31,35,39,41,45)^. Three studies identified gaps related to professional performance as a barrier to preventing violence against children^(35,41,45)^. These include: lack of training, knowledge, and confidence in advising parents on child discipline^(35)^; poor familiarity with guidelines and low self-efficacy in situations involving ambiguous signs, caregiver communication, follow-up planning, and interagency information sharing^(41)^; and the need for professional development with access to clinical supervision in supportive learning environments^(45)^.

Contextual barriers were reported in three studies^(35,41,45)^, including: cultural sensitivity concerns, perceived low priority of the topic, discomfort in addressing it, and doubts about the effectiveness of the intervention^(35)^; caregiver unwillingness or inability to cooperate and greater uncertainty when discussing suspected abuse in home settings^(41)^; and psychosocial influences such as burnout, compassion fatigue, and personal factors, exclusion from safeguarding discussions, and a mismatch between standardized practices and family needs^(45)^.

Four studies described organizational and resource constraints^(31,35,41,45)^. These include: reduced numbers of professionals^(31)^; limited time availability, absence of financial reimbursement, and insufficient institutional support^(35)^; flaws in electronic medical records and poor interagency cooperation^(41)^; and high workloads, unrealistic expectations, understaffing, structural and communication gaps, absence of interdepartmental agreements, and limited service availability^(45)^.

### Barriers to Identying Violence Against Children

Twenty-one studies included in this review identify barriers to identifying violence against children^(25,27,28,29,30,31,33,36,38,40,46,48,51,52,54,55,58,59,60,61,65)^.

Eighteen studies identified professional performance gaps as barriers to identifying violence against children^(27,28,29,30,31,33,36,38,40,48,51,52,54,55,58,59,60,61)^. These include: lack of training and awareness^(27,55,59)^; insufficient knowledge, experience, and confidence in detecting signs and symptoms of abuse^(28,33,36,40,48,52,58)^; difficulty distinguishing between accidental and intentional injuries^(28,61)^; low familiarity with guidelines and tools^(29,48)^; inability to recognize behavioral indicators of neglect^(60)^; inadequate communication skills with children and caregivers^(30,33,38)^; poor interview techniques^(58)^; uncertainty in clinical presentations^(48)^; and discomfort with pediatric cases^(61)^.

Fourteen studies reported contextual barriers^(25,27,29,30,33,36,38,46,51,52,58,60,65)^. These include: sensitivity or taboo nature of the topic^(27,28,52)^; concealment or denial by families^(36,46,52)^; socioeconomic factors masking neglect^(46)^; diffusion of responsibility^(25)^; uncertainty in screening interpretation^(29)^; perception that tools are unnecessary^(30)^; language and cultural barriers^(33)^; professionals’ reluctance to believe abuse occurs^(52,65)^; desire to believe caregivers^(60)^; personal prejudices^(60)^; fear of reporting consequences^(65)^; fear of incorrect identification^(38,58)^; fear of harm^(38)^; and challenges of emergency settings such as lack of ongoing family contact^(60)^.

Sixteen studies described organizational and resource constraints^(27,28,29,30,31,33,36,38,48,51,52,55,58,59,61,65)^. These include: lack of organizational policy or guidelines^(27,51)^; limited or absent resources^(27,55,59)^; insufficient staffing or time^(29,30,55,59)^; poor interdepartmental or interagency coordination^(29,48)^; delays in consultations^(29)^; inadequate documentation infrastructure^(29)^; absence of validated tools^(48)^; lack of identification protocols ^(33)^; need to strengthen primary healthcare as a point of reference^(31)^; heavy workloads^(36,38,59)^; and lack of confidence in social services’ ability to manage cases^(65)^.

### Barriers to Reporting Violence Against Children

Twenty-seven studies included in this review identify barriers to reporting violence against children^(9,19,24,25,26,28,31,32,37,38,39,40,43,47,48,49,50,53,54,55,56,57,58,61,63,64,65)^.

Professional performance gaps were identified in 21 studies^(9,19,25,26,28,31,32,37,38,40,47,48,49,53,54,55,56,58,63,64,65)^. Reported issues include insufficient knowledge or skills in identification and reporting procedures^(9,19,25,31,32,37,38,47,48,49,54,55,58,63,64)^, difficulty preparing reports^(38,54)^, and lack of confidence or experience^(9,28,64)^. Other barriers include subjective decision-making^(58)^, uncertainty about what should be reported^(47,58)^, and the belief in being able to intervene more effectively without formal reporting^(32)^.

Contextual barriers were noted in twenty-five studies^(9,19,24,25,26,28,29,31,32,37,40,43,46,47,48,49,53,54,55,56,57,58,61,63,64,65)^. These include: fear of physical or spiritual harm^(9,24,53,54,64)^; fear of incorrect identification^(54,58,63)^; stigma and cultural or religious norms^(9,19,40,64)^; lack of corroborating evidence^(26,32,43)^; and concerns about the negative consequences of reporting for the professional, the child, or the family^(37,43,47,49,58,61,63,65)^. Additional barriers include distrust in child protection services^(64,65)^, reluctance to engage with legal systems^(43,49)^, belief others will report^(47)^, emotional challenges^(19)^, and hierarchical or interprofessional dynamics that discourage reporting^(28,58)^.

Organizational and resource constraints were reported in 23 studies^(9,19,25,26,28,29,31,37,39,43,46,47,48,49,50,54,55,56,57,58,61,64,65)^. Reported barriers include: lack of organizational policies, clear guidelines, or structured protocols^(19,26,31,58)^; insufficient time and high workloads^(9,25,28,37,43,47,48,49,54,55,57,58,61,64)^; inadequate resources or staffing^(19,31,48,50)^; poor interagency communication or feedback^(19,39,50,56)^; and procedural or administrative barriers^(54,58,64)^. Other limitations include ineffective training implementation^(9)^, absence of legal consequences for non-reporting^(9)^, and lack of protection for reporters^(19)^.

### Barriers to Intervening in Violence Against Children

Nine studies included in this review identify barriers to intervening in violence against children^(18,27,31,34,39,42,44,56,62)^.

Professional performance gaps were reported in six studies^(18,27,34,42,56,62)^. These include: lack of training and awareness about specific forms of violence such as sex trafficking^(27)^; insufficient knowledge in general professional practice^(34)^; subjective decision-making and lack of adequate training^(42)^; gaps between theoretical knowledge and practical application as well as lack of support in formal procedures^(56)^; difficulty defining and identifying negligence; insufficient training; and inability to use toolkits due to high staff turnover^(18)^.

Contextual barriers were identified in six studies^(18,27,34,39,42,44)^. These include: sensitivity of the topic and victims’ fear of reporting^(27)^; cultural factors influencing professional action^(34,42)^; concerns that monitoring families “at risk” could harm the nurse’s friendly image^(39)^; fear of making mistakes and dealing with inflexible systems^(44)^; and situations where children’s opinions are not considered or where professionals adhere to an “optimism rule”^(18)^.

Organizational and resource constraints were mentioned in eight studies^(18,27,31,34,42,44,56,62)^. These include: lack of organizational policies and guidance, and insufficient financing or resources^(27)^; poor network performance due to sectoral and vertical structures and unbalanced communication^(31)^; lack of resources and organizational capacity, implementation challenges, and inadequate working conditions for frontline staff^(34)^; scarce resources, absence of multidisciplinary collaboration, lack of clear protocols, limited autonomy, and hierarchical structures^(42)^; poor interconnection of services, duplication, poor coordination, rigid systems, and information-sharing hierarchies^(44)^; lack of information about case developments^(56)^; limited resources, high staff turnover, excessive caseloads affecting child protection plan management, and lack of supervision^(18)^; and variability in institutional support for champions, reliance on individual motivation, role overload, rotation of champions, and inconsistent program knowledge among front-line providers^(62)^.

## CONCLUSION

This scoping review identified multiple and interconnected barriers faced by healthcare professionals in the prevention, identification, reporting, and intervention in cases of violence against children. These barriers fall into three main dimensions: gaps in professional performance; contextual challenges; and organizational and resource-related constraints. The limitations identified include deficits in knowledge and training, uncertainty about legal procedures, the influence of cultural norms, absence of clear protocols, and insufficient institutional support.

Despite the breadth of the evidence analyzed, this review presents methodological limitations, including the absence of quality appraisal of included studies and methodological heterogeneity, which may have constrained the identification of relevant nuances. However, in accordance with JBI recommendations for scoping reviews, the critical appraisal of methodological quality is not mandatory, as the primary aim is to map the extent and nature of available evidence rather than to assess intervention effectiveness. Moreover, as the searches were conducted within a defined time window and were not continuously updated, relevant studies published after the search period may not have been captured. Although a comprehensive search strategy across multiple databases was implemented, the possibility of missing eligible studies cannot be excluded. Furthermore, although the eligibility criteria encompassed a broad range of healthcare and allied professionals involved in child protection, the search strategy primarily employed terms related to healthcare personnel and pediatric nursing. This may have limited the retrieval of studies involving professionals from other fields, such as social or human sciences, potentially restricting the breadth of professional perspectives captured. Variations in study design, professional groups, and contextual settings also introduced substantial heterogeneity, which may have influenced the synthesis of results. These factors should be taken into account when interpreting the findings of this scoping review.

Nevertheless, despite methodological and contextual differences, several barriers emerged consistently across the literature, suggesting that certain structural and professional challenges are pervasive and may constitute global obstacles to the effective prevention, identification, reporting, and intervention in cases of violence against children. This diversity enriches the understanding of how such barriers manifest in different health systems and sociocultural contexts, supporting the development of context-sensitive strategies for professional capacity-building and policy formulation.

Investment in continuing and specific training, improved working conditions, and stronger inter-institutional collaboration are recommended. Future research should focus on developing and assessing interventions that enhance professionals’ response capacity, particularly by assessing the impact of training programs and collaborative strategies adapted to different cultural and geographic contexts. In addition, greater attention should be given to community-based interventions and longitudinal research designs, which may help to capture how barriers to preventing violence against children evolve over time, as well as to assess the sustainability and long-term effectiveness of interventions across different settings.

## Data Availability

The entire dataset supporting the results of this study is available upon request to the corresponding author.
