# Supplementary material for: Barriers to the prevention of violence against children identified by healthcare professionals: a scoping review
Source: Rev Esc Enferm USP. 2026 Jun 1;60:e20250375. doi: 10.1590/1980-220X-REEUSP-2025-0375en (PMC13228764; doi:10.1590/1980-220X-REEUSP-2025-0375en)
Supplement: Translation

# Translation: Barreras a la prevención de la violencia contra los niños identificadas por profesionales de la salud: una revisión de alcance

**DOI:** 10.1590/1980-220X-REEUSP-2025-0375es

**Authors:** Joana Rita Guarda da Venda Rodrigues, Sílvia Vinagre Luz, Susana Margarida Rodrigues Custódio, Ana Isabel dos Santos Pastorinho, Susana Queiroz Santos, Ana Sofia Lopes Caldeira, Juan Diego Ramos-Pichardo, Maria do Céu Barbieri-Figueiredo

**Affiliations:** 1 Centro de Investigação Inovação e Desenvolvimento em Enfermagem de Lisboa (CIDNUR), Escola Superior de Enfermagem, Universidade de Lisboa, Lisboa, Portugal.; 2 Escola Superior de Enfermagem da Universidade de Lisboa, Lisboa, Portugal.; 3 Hospital Dona Estefânia, Unidade Local de Saúde de São José, Cirurgia Pediátrica e Unidade de Queimados, Lisboa, Portugal.; 4 Escola Superior de Saúde, Politécnico de Leiria, Leiria, Portugal.; 5 Centro de Inovação em Tecnologias e Cuidados da Saúde (ciTechCare), Politécnico de Leiria, Leiria, Portugal.; 6 Hospital Dona Estefânia, Unidade Local de Saúde de São José, Serviço de Urgência Pediátrica Polivalente, Lisboa, Portugal.; 7 Hospital de São Francisco Xavier, Unidade Local de Saúde Lisboa Ocidental, Pediatria, Lisboa, Portugal.; 8 Universidad de Huelva, Faculdad de Enfermería, Huelva, España.; 9 Grupo de Investigación HIGIA (CTS-500), Universidad de Huelva, España.; 10 Universidade do Porto, Escola Superior de Enfermagem, RISE-Health, Porto, Portugal.

**Keywords:** Maltrato a los Niños, Maltrato Infantil, Personal de Salud, Familia, Revisión

## Abstract

**Objetivo:**

Identificar las barreras identificadas por los profesionales sanitarios para la prevención de la violencia contra la infancia.

**Método:**

Esta revisión del alcance se realizó siguiendo la metodología del JBI para revisiones del alcance. La búsqueda se realizó en las bases de datos MedLine, CINAHL, Psychology and Behavioral Sciences Collection, ERIC, Cochrane, MedicLatina, Scopus, Web of Science, RCAAP y MedNar.

**Resultados:**

Se identificaron 1.674 publicaciones, de las cuales 45 estudios se incluyeron en la revisión. Las barreras más frecuentes se relacionaron con la identificación y la denuncia de la violencia contra la infancia. Entre los desafíos recurrentes se encontraban las deficiencias en conocimientos y formación, la inseguridad profesional, la sobrecarga de trabajo y la disponibilidad limitada de tiempo. Los factores culturales, sociales y organizativos también surgieron como obstáculos transversales significativos.

**Conclusión:**

La necesidad de capacitación específica, mejores condiciones de trabajo y una colaboración interinstitucional fortalecida se destaca como un área prioritaria identificada en la literatura para abordar las barreras complejas e interconectadas que enfrentan los profesionales de la salud en la prevención y el combate de la violencia contra los niños.

## INTRODUCCIÓN

La violencia contra los niños abarca todas las formas de violencia infligida a personas menores de 18 años^(1)^. Aproximadamente 400 millones de niños pequeños en todo el mundo —lo que equivale a seis de cada diez niños menores de 5 años— sufren regularmente castigos físicos y/o violencia psicológica a manos de sus padres y cuidadores^(2,3)^.

Según la Convención sobre los Derechos del Niño, el uso de la violencia contra los niños constituye una violación de sus derechos^(4)^. Reconociendo la gravedad de este problema, como parte de la Agenda 2030 para el Desarrollo Sostenible, la meta 16.2 tiene como objetivo eliminar el abuso, la explotación, la trata y todas las formas de violencia y tortura dirigidas contra los niños^(5)^.

Una gran proporción de la violencia contra los niños implica diversas formas de violencia interpersonal, que tienden a ocurrir en diferentes etapas del desarrollo infantil. El maltrato infantil es un tipo de violencia contra los niños que implica abuso y negligencia. Incluye «todos los tipos de maltrato físico y/o emocional, abuso sexual, abandono, negligencia y explotación comercial o de otro tipo, que resulten en un daño real o potencial a la salud, la supervivencia, el desarrollo o la dignidad del niño en el contexto de una relación de responsabilidad, confianza o poder»^(3)^. La evidencia de diversos contextos geográficos y culturales muestra que la violencia contra los niños es prevenible^(1)^.

Sufrir violencia durante la infancia tiene consecuencias profundas y duraderas para la salud y el bienestar, lo que afecta negativamente al desarrollo físico, emocional, social y psicológico a lo largo de toda la vida^(1,6,7)^. La violencia merma la capacidad del niño o adolescente para comprender, expresar y reconocer sus emociones, lo que puede conducir al aislamiento social y, en consecuencia, a un mayor riesgo de conductas adictivas y de alto riesgo, depresión, ansiedad e ideas suicidas^(8,9)^.

Existen numerosos factores de riesgo asociados al maltrato infantil, como la edad del niño, la presencia de problemas de salud y/o de conducta, expectativas parentales poco realistas, embarazos no planificados y antecedentes familiares de violencia^(10)^. Por el contrario, también se han identificado factores de protección, como la práctica de una crianza positiva, la presencia de un apego seguro entre el niño y su familia, una red eficaz de apoyo familiar y social, y el fácil acceso a los servicios comunitarios, incluidos los servicios sociales, educativos y de salud^(10,11,12)^. En 2016, reconociendo el impacto devastador de la violencia, el Secretario General de las Naciones Unidas lanzó la Alianza Mundial para Poner Fin a la Violencia contra los Niños^(11)^.

Por lo tanto, debido a su estrecho contacto tanto con el niño como con la familia, los profesionales de la salud desempeñan un papel central en la prevención, la intervención y la denuncia del maltrato infantil^(1,11)^. Las disciplinas clave incluyen enfermeras, parteras, trabajadores sociales, psicólogos, terapeutas ocupacionales, fisioterapeutas, logopedas y profesionales médicos, como médicos generales, pediatras y psiquiatras^(13)^.

Se ha demostrado que la intervención de estos profesionales influye en el desarrollo y el bienestar de los niños^(14)^, lo que refuerza la importancia de garantizar que estén adecuadamente preparados para defender los derechos y la protección de los niños y adolescentes^(1,15)^. Sin embargo, la evidencia indica que, a pesar de su alta prevalencia, la violencia contra los niños a menudo permanece oculta, invisible o sub-notificada^(9,10)^, lo que puede comprometer la eficacia de las intervenciones en este complejo fenómeno^(9)^. Los estudios muestran que los profesionales de la salud a menudo se sienten poco preparados para abordar estos casos debido a la falta de conocimientos y habilidades adecuados^(16,17)^. Varios estudios enfatizan la importancia de capacitar a los proveedores en temas de maltrato infantil y violencia contra los niños^(15)^. Aunque ya se han identificado algunas barreras que afectan la capacidad de los profesionales de la salud para prevenir, identificar, intervenir y denunciar casos de violencia contra los niños, estas se encuentran dispersas en la literatura. En diferentes contextos globales, estas barreras van más allá de las lagunas de conocimiento individuales e incluyen altas cargas de trabajo, disponibilidad de tiempo limitada, procedimientos institucionales y legales poco claros, apoyo organizacional insuficiente, normas culturales y coordinación intersectorial fragmentada^(15,16,17)^. Estos desafíos pueden limitar la capacidad de los profesionales de la salud para prevenir la violencia contra los niños en su práctica diaria, a pesar de la reconocida relevancia de su intervención en la protección infantil.

Se realizó una búsqueda preliminar en las bases de datos MedLine, Cochrane Database of Systematic Reviews, JBI Evidence Synthesis y Open Science Framework, y no se identificaron revisiones sistemáticas ni revisiones exploratorias actuales o en curso sobre el tema, específicamente en lo que respecta a la identificación de barreras para la prevención y la respuesta a la violencia contra los niños por parte de los profesionales de la salud. Aunque existen revisiones previas sobre temas relacionados, su alcance sigue siendo limitado en términos de cobertura profesional, conceptual o geográfica. Solem et al.^(18)^ se centraron exclusivamente en las barreras a las que se enfrentan los trabajadores sociales en Inglaterra a la hora de identificar y responder a casos de negligencia infantil, mientras que Wilson y Lee^(19)^ analizaron los factores asociados con los comportamientos de denuncia obligatoria. Owaidah et al.^(9)^, por su parte, exploraron los factores que influyen en la falta de denuncia del maltrato infantil en Arabia Saudita. En este contexto, la presente revisión de alcance se distingue por su objetivo de mapear de manera integral e interprofesional las barreras identificadas por los profesionales de la salud en la prevención y la respuesta a la violencia contra los niños, con especial énfasis en las dimensiones de prevención, identificación y denuncia. Al proporcionar una visión general exhaustiva y actualizada de la evidencia internacional, esta revisión puede ayudar a identificar lagunas en la literatura existente, respaldar una respuesta más informada e integrada al fenómeno de la violencia contra los niños y orientar estrategias futuras en materia de formación, práctica profesional e investigación. En consecuencia, busca responder a la siguiente pregunta de revisión: ¿cuáles son las barreras para prevenir la violencia contra los niños identificadas por los profesionales de la salud? Además, pretende abordar la sub-pregunta: ¿cuáles son las barreras para la identificación, la denuncia y la intervención en situaciones de violencia contra los niños identificadas por los profesionales de la salud? En consonancia con estos objetivos, esta revisión tiene como objetivo mapear las barreras identificadas por los profesionales de la salud en la prevención de la violencia contra los niños.

## MÉTODO

Esta revisión de alcance se llevó a cabo siguiendo la metodología recomendada por el JBI^(20)^ y de acuerdo con la lista de verificación de los Elementos Preferidos para Informes de Revisiones Sistemáticas y Metaanálisis: extensión para revisiones exploratorias (PRISMA-ScR)^(21,22)^, asociada al diagrama de flujo PRISMA 2020^(23)^. El protocolo de la revisión fue redactado y analizado por los autores, y registrado en el Open Science Framework (https://osf.io/8nwx7/).

### Criterios de Elegibilidad

Los criterios de elegibilidad de los estudios se definieron basándose en el acrónimo PCC (Población, Concepto y Contexto). Se incluyeron los estudios en los que la población estaba compuesta por profesionales de la salud, tales como enfermeras, parteras, trabajadores sociales, psicólogos, terapeutas ocupacionales, fisioterapeutas, logopedas y profesionales médicos —como médicos generales, pediatras y psiquiatras—. En cuanto al concepto, esta revisión examina los estudios que abordan las barreras para prevenir y combatir situaciones de violencia contra los niños identificadas por los profesionales de la salud. Se consideraron todos los entornos de atención médica, sin ninguna limitación. Esta revisión incluyó estudios de tipo cualitativo, cuantitativo o mixto, así como revisiones de literatura y literatura gris. Se consideraron documentos en todos los idiomas, sin restricciones en cuanto a la fecha de publicación. Se excluyeron los estudios que no se centraban explícitamente en la violencia contra los niños.

### Fuentes de Información

Para identificar los estudios, se utilizaron las bases de datos electrónicas Medical Literature Analysis and Retrieval System Online (MedLine Ultimate) (a través de EBSCO), Cumulative Index of Nursing and Allied Health Literature (CINAHL Ultimate) (a través de EBSCO), Psychology and Behavioral Sciences Collection, ERIC, Cochrane Central Register of Controlled Trials, MedicLatina, Scopus y Web of Science. Para identificar estudios no publicados/literatura gris, se realizó una búsqueda en el *Repositório Científico de Acesso Aberto de Portugal* (RCAAP) y en MedNar. El proceso de búsqueda abarcó tres etapas. En la primera etapa, las búsquedas se realizaron en la plataforma web EBSCOhost, y se utilizaron las bases de datos electrónicas MedLine Ultimate y CINAHL Ultimate. Se utilizaron términos de búsqueda en lenguaje natural para identificar las palabras clave utilizadas en los títulos y resúmenes, así como los términos de indexación. En la segunda etapa, las palabras naturales, las palabras clave y los términos de indexación enumerados se combinaron con operadores booleanos y, cuando fue posible, con un operador asterisco (*), para formar la expresión de búsqueda, que se adaptó a las especificidades de cada base de datos o repositorio. Los términos combinados utilizados incluyeron: barrier* OR difficult* OR “child abuse prevention” OR “child abuse intervention” OR “child abuse control” AND “Health Personnel+” OR “Pediatric Nurse Practitioners+” AND “violence against child*” OR “Child Abuse” OR “child maltreatment” OR mistreatment OR violence (Cuadro 1). La búsqueda se llevó a cabo entre julio y octubre de 2024. En la tercera etapa, se analizaron las referencias bibliográficas de los registros identificados anteriormente. También se buscaron estudios no publicados y literatura gris en los repositorios de las bibliotecas.

**Cuadro 1:** Estrategias de búsqueda para la revisión de alcance – Lisboa, Portugal, 2024.

Base de datos/ fechas de búsqueda	Estrategia de búsqueda	Resultados
CINAHL Ultimate (via EBSCO) July, 2024	(TI ( barrier* OR difficult* OR child abuse prevention OR child abuse intervention” OR child abuse control) OR AB (barrier* OR difficult* OR child abuse prevention” OR child abuse intervention OR child abuse control”)) AND (MH “Health Personnel+”) OR (MH “Pediatric Nurse Practitioners+”) AND (TI ( child maltreatment OR mistreatment OR violence against child*) OR AB (child maltreatment OR mistreatment OR violence against child*) OR (MH “Child Abuse”) OR (MH “Violence”))	284
MedLine Ultimate (via EBSCO) July, 2024	((((((“barrier*” [Title/Abstract]) OR “difficult*” [Title/Abstract]) OR “child abuse prevention” [Title/Abstract]) OR “child abuse intervention” [Title/Abstract]) OR “child abuse control” [Title/Abstract]) AND (((“Health Personnel+”[Mesh]) OR “Pediatric Nursing+”[Mesh]) AND ((((((“child maltreatment” [Title/Abstract]) OR “mistreatment” [Title/Abstract]) OR “violence against child*”[Title/Abstract]) OR (“Child Abuse”[Mesh])) OR (“Violence”[Mesh]))	355
Scopus October, 2024	(TITLE-ABS-KEY ( barrier* ) OR TITLE-ABS-KEY ( difficult* ) OR TITLE-ABS-KEY ( “child abuse prevention” ) OR TITLE-ABS-KEY ( “child abuse intervention” ) OR TITLE-ABS-KEY ( “child abuse control” ) AND TITLE-ABS-KEY ( “Health Personnel” ) OR TITLE-ABS-KEY ( “Pediatric Nurse Practitioners” ) AND TITLE-ABS-KEY ( “child maltreatment”) OR TITLE-ABS-KEY ( mistreatment ) OR TITLE-ABS-KEY ( “violence against child*” ) OR TITLE-ABS-KEY (“Child Abuse”) OR TITLE-ABS-KEY ( violence ) )	649
Psychology and Behavioral Sciences Collection July, 2024	(TI ( barrier* OR difficult* OR “child abuse prevention” OR “child abuse intervention” OR “child abuse control”) OR AB ( barrier* OR difficult* OR “child abuse prevention” OR “child abuse intervention” OR “child abuse control” ) AND (TI (“Health Personnel” OR “Pediatric Nurse Practitioners” ) OR AB ( “Health Personnel” OR “Pediatric Nurse Practitioners”)) AND ( TI (“child maltreatment” OR mistreatment OR “violence against child*” OR “Child Abuse” OR violence) OR AB (“child maltreatment” OR mistreatment OR “violence against child*” OR “Child Abuse” OR violence))	0
Cochrane Central Register of Controlled Trials July, 2024	(TI ( barrier* OR difficult* OR “child abuse prevention” OR “child abuse intervention” OR “child abuse control”) OR AB ( barrier* OR difficult* OR “child abuse prevention” OR “child abuse intervention” OR “child abuse control”)) AND (TI ( “Health Personnel” OR “Pediatric Nurse Practitioners” ) OR AB ( “Health Personnel” OR “Pediatric Nurse Practitioners”)) AND ( TI ( “child maltreatment” OR mistreatment OR “violence against child*” OR “Child Abuse” OR violence) OR AB (“child maltreatment” OR mistreatment OR “violence against child*” OR “Child Abuse” OR violence ) )	0
MedicLatina July, 2024	(TI ( barrier* OR difficult* OR “child abuse prevention” OR “child abuse intervention” OR “child abuse control” ) OR AB ( barrier* OR difficult* OR “child abuse prevention” OR “child abuse intervention” OR “child abuse control” ) ) AND (TI ( “Health Personnel” OR “Pediatric Nurse Practitioners” ) OR AB ( “Health Personnel” OR “Pediatric Nurse Practitioners” ) ) AND ( TI ( “child maltreatment” OR mistreatment OR “violence against child*” OR “Child Abuse” OR violence ) OR AB ( “child maltreatment” OR mistreatment OR “violence against child*” OR “Child Abuse” OR violence ) )	0
Web of Science October, 2024	“child maltreatment” AND “health personnel”	7
RCAAP October, 2024	“child maltreatment”	65
CAPES October, 2024	TI (“child maltreatment” AND “health personnel”)	4
MedNar October, 2024	TI (“child maltreatment”) Limit to Available Full-Text	325

### Extracción de Datos

Los resultados de la búsqueda se exportaron al gestor de referencias Mendeley Desktop (versión 1.19.8), donde se identificaron y eliminaron los registros duplicados. Posteriormente, para facilitar el proceso de selección, los registros se importaron a la plataforma del Instituto de Investigación Informática de Qatar (Rayyan QCRI), que se utilizó para organizar los registros y facilitar la selección ciega. La elegibilidad fue evaluada por dos revisores independientes que examinaron los títulos y los resúmenes según los criterios de inclusión y exclusión predefinidos. La elegibilidad fue evaluada por dos revisores independientes que examinaron los títulos y los resúmenes según los criterios de inclusión y exclusión predefinidos, y las discrepancias se resolvieron mediante discusión o consulta con un tercer revisor. Los artículos que cumplían con los criterios de elegibilidad se recuperaron en su totalidad, y el texto completo fue evaluado de forma independiente y detallada por dos o más revisores para determinar su elegibilidad.

### Resumen de los Resultados

La síntesis de datos de los registros que cumplían los criterios de inclusión y exclusión fue realizada de forma independiente por dos autores, utilizando instrumentos de extracción de datos desarrollados por el equipo de investigación, en consonancia con los objetivos y la pregunta de esta revisión de alcance. La información extraída se introdujo en una hoja de cálculo de Microsoft Excel. Los resultados se presentan en tablas resumidas.

Tras evaluar los registros de cada uno, los autores destacaron los aspectos que necesitaban aclaración. Estas inquietudes se abordaron mediante debate y se presentaron los resultados.

## RESULTADOS

Se identificaron un total de 1674 artículos en las bases de datos, de los cuales se eliminaron 44 por ser duplicados. Tras la selección de títulos y resúmenes, se seleccionaron 105 artículos para la evaluación del texto completo. Se utilizó la metodología PRISMA-ScR^(23)^ (Figura 1) para sistematizar el proceso de inclusión de los estudios. Las Cuadros 2, 3, 4, 5 y 6 presentan un resumen de los principales hallazgos. Los registros excluidos durante la selección de títulos y resúmenes no cumplían con los criterios de elegibilidad establecidos para esta revisión, ya que no se centraban en la violencia contra los niños, no involucraban a profesionales de la salud según la definición establecida o no examinaban las barreras en la prevención y la lucha contra la violencia contra los niños identificadas por los profesionales de la salud. En todos los cuadros, los números de los estudios corresponden a las referencias tal como están numeradas en la lista de referencias al estilo de Vancouver.

**Figura 1:** Diagrama de flujo del proceso de búsqueda y selección de estudios.

**Cuadro 2:** Características generales de los estudios revisados – Lisboa, Portugal, 2025.

Estudio (año)	País	Diseño del estudio	Objetivo(s)	Entorno	Tipo de violencia contra los niños
Antwi et al. (2019)^(24)^	Ghana	Cualitativo	Explorar las influencias externas en la toma de decisiones de los radiólogos ghaneses a la hora de denunciar presuntos casos de maltrato físico infantil; identificar las influencias culturales y las creencias relativas a la denuncia de maltrato físico infantil por parte de los radiólogos en Ghana.	Hospital	Maltrato físico
Antwi et al. (2021)^(25)^	Ghana	Cualitativo	Explorar los factores internos que determinan las actitudes de los testigos ante la denuncia de presuntos casos de maltrato físico entre los radiólogos ghaneses.	Hospital	Abuso físico
Beck y Ogloff (1995)^(26)^	Canadá	Método mixto	Investigar factores específicos relacionados con la denuncia de maltrato infantil, controlando algunas de las limitaciones de investigaciones anteriores.	Directorio de la Facultad de Psicología	Maltrato físico Maltrato emocional Abuso sexual Negligencia
Beck et al. (2015)^(27)^	Estados Unidos de América	Método mixto	Evaluar las lagunas de conocimiento y las necesidades de formación de los profesionales sanitarios; demostrar la importancia de la formación de los profesionales para satisfacer las necesidades específicas de las víctimas pediátricas de la trata con fines sexuales; y poner de relieve las barreras para identificar y atender a las víctimas.	Hospital	Trata sexual
Berchtold et al. (2023)^(28)^	Suiza	Método mixto	Examinar los obstáculos y los factores que facilitan la detección y la denuncia del maltrato infantil entre el personal de enfermería y médico de los servicios de urgencias pediátricas y de cirugía pediátrica.	Hospital	Maltrato infantil
Carson (2018)^(29)^	Estados Unidos de América	Proyecto de mejora de la calidad	Implementar un programa de detección basado en la evidencia que incluya formación para los profesionales sobre el abuso físico y de menores, un protocolo de detección sistemática y el uso del instrumento Escape validado.	Servicio de Urgencias Pediátricas	Maltrato físico
Crichton et al. (2016)^(30)^	Colombia	Cualitativo	Evaluar el uso y las características de los protocolos de detección de maltrato físico en los hospitales infantiles y determinar las actitudes hacia el uso de dichas herramientas.	Hospital pediátrico	Maltrato físico
Egry et al. (2017)^(31)^	Brasil	Cualitativo	Comprender la percepción de los profesionales sanitarios que trabajan en atención primaria sobre la violencia infantil.	Comunitario servicios de salud	
Elarousy y Abed (2019)^(32)^	Arabia Saudí	Descriptivo	Investigar las barreras que impiden que el personal de enfermería de la Ciudad Médica Rey Abdulaziz, en Yeda (Arabia Saudí), notifique los casos sospechosos de maltrato y negligencia infantil.	Hospital pediátrico	Maltrato infantil Descuido
Eniola y Evarts (2017)^(33)^	Estados Unidos de América	Método mixto	Ofrecer recomendaciones para mejorar la confianza de los médicos de cabecera a la hora de diagnosticar el maltrato infantil.	Academia Americana de Médicos de Familia	
Faryan et al. (2019)^(34)^	Arabia Saudí	Fenomenológico	Explora las experiencias de los trabajadores de protección infantil en Arabia Saudí.		Maltrato infantil Descuido
Fleckman et al. (2021)^(35)^	Estados Unidos de América	Método mixto	Evaluar la preparación, la motivación y las barreras de los pediatras estadounidenses en relación con la educación de los padres sobre el castigo físico y la disciplina infantil eficaz.	Base de datos nacional de Estados Unidos	Abuso físico y emocional
Galindo et al. (2017)^(36)^	Brasil	Cualitativo	Analizar los conocimientos de las enfermeras sobre los tipos de violencia infantil y adolescente, identificando las conductas, las dificultades y las posibles acciones de prevención y promoción de la salud diseñadas en las Estrategias de Salud Familiar.	Servicios de salud comunitarios	Abuso sexual Violencia física Descuido
Gunn et al. (2005)^(37)^	Estados Unidos de América	Método mixto	Identificar los factores asociados a la decisión de los pediatras de no denunciar los casos de sospecha de maltrato infantil.	Departamento de Salud	Maltrato infantil Negligencia
İnanici et al. (2020)^(38)^	Turquía	Método mixto	Investigar las experiencias de los médicos con casos de maltrato, sus evaluaciones de los factores de riesgo de maltrato y sus actitudes respecto a recibir más formación sobre el tema.	Servicios de salud comunitarios	Maltrato infantil Descuido
Kent et al. (2011)^(39)^	Irlanda	Cualitativo	Explorar las opiniones de un grupo de enfermeras de salud pública sobre su papel con los niños en edad preescolar.	Servicios de atención sanitaria comunitaria	
Khanjari et al. (2021)^(40)^	Irán	Cuasi-experimental	Determinar el efecto de un programa educativo en el reconocimiento y la intención de las enfermeras iraníes de denunciar casos de maltrato infantil.	Servicio de urgencias pediátricas y planta de pediatría	
Konijnendijk et al. (2014)^(41)^	Países Bajos	Cualitativo	Identificar los factores relacionados con las características de las directrices, el usuario, la organización y el contexto sociopolítico que facilitan o dificultan el cumplimiento de las directrices de prevención del maltrato infantil.	Organización para la Salud Infantil	Maltrato infantil
Land y Barclay (2008)^(42)^	Australia	Cualitativo	Explorar, con una pequeña muestra de enfermeras de salud infantil, pediátricas y escolares, su comprensión y conocimiento sobre el maltrato y el abandono infantil; identificar las percepciones de estas enfermeras sobre su papel y su implicación en la protección de los niños; e identificar cualquier barrera percibida que impida a las enfermeras desempeñar un papel en la protección de los niños.	Servicios de salud comunitarios	Maltrato infantil Descuido
Leung et al. (2011)^(43)^	Hong Kong	Estudio transversal	Investigar las actitudes y comportamientos de los médicos generales de Hong Kong respecto a la denuncia del maltrato infantil y sus opiniones sobre la introducción de un sistema de denuncia obligatoria.		Maltrato infantil
Lines et al. (2020)^(44)^	Australia	Cualitativo	Informar sobre las percepciones de las enfermeras respecto a cómo los sistemas y jerarquías organizativos condicionaban su capacidad para responder al maltrato y el abandono infantil.	Servicios de salud comunitarios y hospital	Maltrato infantil Descuido
Lines et al. (2024)^(45)^	Australia	Cualitativo	Desarrollar una comprensión más amplia y completa de las barreras a las que se enfrentan las enfermeras y las matronas a la hora de proteger a los niños.	Entornos centrados en el niño	
Lobato et al. (2012)^(46)^	Brasil	Estudio de caso	Analizar la percepción de los profesionales de los equipos de salud familiar sobre la violencia en su ámbito de actividad y los retos a la hora de notificar casos y prestar atención a las familias en situaciones de violencia.	Servicios de salud comunitarios	Violencia doméstica contra niños y adolescentes Maltrato físico Descuido Abandono
Lynne et al. (2015)^(47)^	Estados Unidos de América	Método mixto	Comprender por qué los proveedores de servicios médicos de urgencias pueden dejar de notificar los casos de sospecha de maltrato a pesar de las políticas de notificación obligatoria.	Servicio de urgencias general	
Mandadi et al. (2021)^(48)^	Estados Unidos de América	Estudio transversal	Describir los conocimientos, la formación, la confianza y las barreras de los médicos de urgencias pediátricas a la hora de reconocer y notificar los casos de sospecha de maltrato infantil.	Urgencias pediátricas	Maltrato infantil Negligencia
Mostovoy et al. (2024)^(49)^	Israel	Cuantitativo	Analizar las actitudes, los conocimientos y la disposición de los oftalmólogos a denunciar el maltrato infantil.	Asociación de Oftalmólogos	Maltrato infantil
Nouman et al. (2020)^(50)^	Israel	Cualitativo	Investigar las diferentes formas en que los profesionales sanitarios que trabajan en servicios comunitarios gestionan la notificación obligatoria, sus fundamentos y su experiencia con las exigencias formales.	La mayor organización de gestión sanitaria	Maltrato infantil Negligencia
Owaidah et al. (2022)^(9)^	Arabia Saudí	Revisión	Determinar los diferentes factores que influyen en la falta de denuncia de los casos de maltrato infantil en Arabia Saudí.		Maltrato infantil Descuido
Paavilainen et al. (2002)^(51)^	Finlandia	Método mixto	Determinar si el personal de enfermería pediátrica y los médicos de un hospital universitario necesitan formación adicional para identificar el maltrato físico infantil y si existe la necesidad de desarrollar programas de educación sanitaria sobre cuestiones relacionadas con el maltrato infantil.	Hospital universitario	Maltrato infantil Maltrato físico Maltrato psicológico Abuso sexual Negligencia
Paavilainen et al. (2002)^(52)^	Finlandia	Cuantitativo	Observar cómo las enfermeras y los médicos de un hospital universitario evaluaban su capacidad para identificar el abuso infantil mientras atendían a estos niños.	Hospital universitario	
Patrick et al. (2020)^(53)^	Reino Unido	Auditoría	Identificar y abordar las barreras que impiden que el personal hospitalario denuncie casos de maltrato infantil.	Hospital	Maltrato infantil Descuido
Regnaut et al. (2015)^(54)^	Francia	Método mixto	Evaluar el nivel de conocimiento que tienen los médicos de familia sobre los factores de riesgo del maltrato infantil y la atención que prestan a dichos factores.	Clínica privada	Maltrato infantil
Riese et al. (2014)^(55)^	Estados Unidos de América	Epidemiológico	Examinar las actitudes, los comportamientos y las barreras de los médicos residentes respecto a la prevención de la violencia juvenil mientras atienden a jóvenes con lesiones por violencia en el ámbito de cuidados intensivos.	Hospital pediátrico	Violencia juvenil
Rowse (2009)^(56)^	Inglaterra	Cualitativo	Explorar las experiencias de las enfermeras de una unidad de pediatría hospitalaria que participaron directamente en casos de protección infantil, con el fin de comprender sus necesidades de apoyo y sugerir mejoras en la formación y el apoyo.	Hospital pediátrico	
Roy et al. (2022)^(57)^	Reino Unido	Método mixto	Evaluar la viabilidad y la aceptabilidad del modelo IRIS+ para niños.	Hospital	Violencia doméstica y maltrato
Russell et al. (2004)^(58)^	Irlanda del Norte	Método mixto	Evaluar la percepción y la capacidad de los profesionales de atención primaria para reconocer el maltrato físico infantil durante la práctica.	Servicios de salud comunitarios	Maltrato físico
Soh et al. (2023)^(59)^	Australia	Método mixto	Identificar las barreras que impiden a los médicos y al personal de enfermería reconocer y denunciar posibles casos de maltrato infantil.	Hospital	Maltrato físico Negligencia Exposición a la violencia familiar Abuso sexual Abuso emocional
Solem et al. (2020)^(18)^	Inglaterra	Revisiones de casos graves	Analiza las barreras a las que se enfrentan los trabajadores sociales en Inglaterra a la hora de identificar y responder a los casos de negligencia de manera oportuna, adecuada y eficaz.	Repositorio nacional de revisiones de casos	Descuido
Tiyyagura et al. (2015)^(60)^	Estados Unidos de América	Cualitativo	Explorar las experiencias de los profesionales de los servicios de urgencias generales en la detección y notificación del maltrato y el abandono infantil; identificar los obstáculos y los factores que facilitan la detección del maltrato y el abandono infantil en el entorno de urgencias.	Servicios generales de urgencias	Maltrato infantil Descuido
Tiyyagura et al. (2017)^(61)^	Estados Unidos de América	Cualitativo	Explorar las barreras y los factores que facilitan el reconocimiento y la denuncia del maltrato y el abandono infantil por parte de los profesionales de la atención prehospitalaria.	Prehospitalaria	Maltrato infantil Descuido
Tiyyagura et al. (2019)^(62)^	Estados Unidos de América	Cualitativo	Comprender los puntos fuertes y los retos del programa; explorar los factores que influyeron en su implementación.	Centro Médico Académico	Maltrato infantil Descuido
Vilensky et al. (2022)^(63)^	Israel	Cualitativo	Identificar las barreras que impiden a las enfermeras comunitarias de Israel denunciar los casos de maltrato infantil.	Servicios de gestión sanitaria, centros de atención primaria	Maltrato infantil
Wißmann et al. (2019)^(64)^	Alemania	Método mixto	Analizar el comportamiento de los pediatras a la hora de denunciar casos de maltrato y negligencia infantil, así como sus actitudes hacia la obligación de denuncia.	Clínica privada	Maltrato infantil Descuido
Willis y Horner (1987)^(65)^	Estados Unidos de América	Método mixto	Estimar el nivel de sospecha y denuncia de abuso sexual infantil por parte de los médicos de familia; identificar posibles barreras cognitivas, conductuales y experienciales para sospechar y denunciar el abuso sexual infantil.	Facultad de Medicina de la Universidad de Carolina del Este, Departamento de Medicina Familiar	Abuso sexual
Wilson y Lee (2021)^(19)^	Estados Unidos de América	Revisión	Revisar la bibliografía existente centrada en los factores relacionados con las conductas de denuncia obligatoria de los profesionales de la atención infantil ante casos de abuso y negligencia infantil, prestando especial atención a las enfermeras; resumir los resultados de los estudios; ofrecer implicaciones para la práctica clínica.		Abuso infantil Descuido

**Cuadro 3:** Barreras para la prevención de la violencia contra los niños – Lisboa, Portugal, 2025.

Estudio	Barreras para la prevención de la violencia contra los niños
Egry et al.^(31)^	- Reducción del número de profesionales.
Fleckman et al.^(35)^	- Falta de formación, conocimientos y confianza a la hora de abordar la disciplina infantil; - Preocupaciones relacionadas con la sensibilidad cultural, la percepción de que el tema tiene baja prioridad, la incomodidad a la hora de debatir la cuestión y las dudas sobre la eficacia de la intervención; - Disponibilidad de tiempo limitada, ausencia de reembolso económico y apoyo institucional insuficiente.
Kent et al.^(39)^	- Dificultades en la definición del riesgo y elevado volumen de casos.
Konijnendijk et al.^(41)^	- Escasa familiaridad con las directrices y baja autoeficacia en casos ambiguos, comunicación con los cuidadores, planificación del seguimiento e intercambio de información entre organismos; - Falta de cooperación de los cuidadores y mayor incertidumbre al abordar los casos de sospecha de maltrato en el ámbito doméstico; - Deficiencias en las historias clínicas electrónicas y escasa cooperación entre organismos.
Lines et al.^(45)^	- Necesidad de desarrollo profesional y acceso a la supervisión clínica; - Influencias psicosociales (agotamiento, fatiga por compasión, factores personales), exclusión de los debates sobre protección y desajuste entre las prácticas estandarizadas y las necesidades de las familias; - Elevadas cargas de trabajo, expectativas poco realistas, falta de personal, deficiencias estructurales y de comunicación, ausencia de acuerdos interdepartamentales y disponibilidad limitada de servicios.

**Cuadro 4:** Barreras para identificar la violencia contra los niños – Lisboa, Portugal, 2025.

Estudio	Barreras para identificar la violencia contra los niños
Antwi et al.^(25)^	- Difusión de la responsabilidad.
Beck et al.^(27)^	- Falta de formación y concienciación sobre el tráfico sexual; - Delicadeza del tema y miedo de las víctimas a denunciar; - Ausencia de políticas o directrices y financiación o recursos insuficientes.
Berchtold et al.^(28)^	- Falta de experiencia, conocimientos y confianza para detectar signos y síntomas de maltrato infantil, y dificultad para distinguir entre sucesos accidentales y no accidentales; - Respeto por el carácter tabú del tema; - Falta de transparencia en el proceso de denuncia.
Carson^(29)^	- El escaso conocimiento del personal de triaje sobre el instrumento Escape; - La incertidumbre sobre los detalles del programa de triaje y los falsos positivos en las pruebas del Escape; - La configuración del servicio de urgencias, las restricciones de tiempo, los retrasos en el equipo de maltrato infantil, las consultas y la ausencia de un lugar específico para documentar los resultados de la detección.
Crichton et al.^(30)^	- Falta de comprensión o concienciación sobre el maltrato infantil y dificultad para comunicarse con los cuidadores en casos sospechosos; - Percepción de que las herramientas de detección son innecesarias; - Falta de tiempo para desarrollar o completar las herramientas de detección, apoyo administrativo insuficiente y recursos comunitarios limitados.
Egry et al.^(31)^	- Confusión entre el abandono y las necesidades básicas insatisfechas debido a la pobreza; - Necesidad de reforzar la atención sanitaria básica como referencia y apoyo para identificar la violencia.
Eniola y Evarts^(33)^	- Inexperiencia, formación inadecuada, falta de confianza en la evaluación de los casos y en la comunicación con los padres; - Barreras lingüísticas y el bagaje cultural de los médicos; - Ausencia de protocolos de identificación y tiempo insuficiente para la exploración física.
Galindo et al.^(36)^	- Lagunas en la formación y las cualificaciones del personal de enfermería; - Información obtenida indirectamente a través de miembros de la comunidad y negación por parte de las familias de la información que respalda las sospechas; - Sobrecarga de tareas para el personal de enfermería.
İnanici et al.^(38)^	- Miedo a identificar incorrectamente el maltrato, dificultad para comunicarse cara a cara con el niño, problemas para controlar las emociones y dificultad para gestionar los procesos psicológicos relacionados con el niño y/o la familia; - Sensación de soledad, miedo a sufrir daños por parte de las personas implicadas e incredulidad ante la posibilidad de que la familia maltrate al niño; - Carga de trabajo.
Khanjari et al.^(40)^	- Bajo nivel de conocimientos de las enfermeras y formación limitada en la detección de signos y síntomas de maltrato infantil.
Lobato et al.^(46)^	- Ocultación de las situaciones por parte de las familias debido a la pobreza, dificultad para identificar actitudes negligentes teniendo en cuenta el contexto socioeconómico.
Mandadi et al.^(48)^	- Falta de conocimientos, incertidumbre ante determinados cuadros clínicos y menor formación sobre el abandono infantil que sobre el maltrato físico; - Disponibilidad de trabajadores sociales, información de las agencias de protección infantil y disponibilidad de herramientas de detección validadas.
Paavilainen et al.^(51)^	- Escasa concienciación sobre el fenómeno; - Carácter inusual del fenómeno; - Ritmo intenso del trabajo en equipo, falta de directrices acordadas conjuntamente.
Paavilainen et al.^(52)^	- Falta de conocimientos y experiencia; - Delicadeza del tema, ocultación por parte de los niños y los padres, explicaciones alternativas para los comportamientos, tendencia a ocultar el abuso, incredulidad ante la veracidad del abuso, diversidad de síntomas; - Presión laboral por parte del equipo.
Regnautet al.^(54)^	- Dificultad para identificar el abuso infantil.
Riese et al.^(55)^	- Falta de formación, falta de confianza en las propias competencias; - falta de tiempo y de recursos hospitalarios y extrahospitalarios.
Russell et al.^(58)^	- Inexperiencia, técnicas de entrevista deficientes; - Miedo a una identificación incorrecta, falta de voluntad para enfrentarse a la familia; - Miedo a que no se mantuviera el anonimato.
Soh et al.^(59)^	- Lagunas en los conocimientos y la formación, falta de comodidad e incertidumbre sobre los procedimientos de notificación; - Falta de tiempo, falta de apoyo por parte del personal directivo, del servicio de asistencia social y de pediatría, dificultad para encontrar directrices, recursos y apoyo limitados, y una carga de trabajo excesiva.
Tiyyagura et al.^(60)^	- Incapacidad para reconocer los indicadores conductuales de maltrato y negligencia; - Deseo de creer a los cuidadores, prejuicios personales, atribución errónea de comportamientos y falta de contacto continuo con la familia en situaciones de emergencia.
Tiyyagura et al.^(61)^	- Incomodidad con los pacientes pediátricos, incertidumbre a la hora de distinguir entre lesiones accidentales e intencionadas, y enfoque en la queja principal; - Oportunidades limitadas para la evaluación.
Willis y Horner^(65)^	- Incapacidad para creer que el abuso se produce con la frecuencia denunciada y creencia de que la denuncia conlleva consecuencias desfavorables; - Falta de confianza en la capacidad de los servicios sociales locales para gestionar el abuso sexual.

**Cuadro 5:** Barreras para denunciar la violencia contra los niños – Lisboa, Portugal, 2025.

Estudio	Barreras para denunciar la violencia contra los niños
Antwi et al.^(24)^	- Miedo a sufrir agresiones físicas o psicológicas, interdependencia cultural y solidaridad.
Antwi et al.^(25)^	- Lagunas de conocimiento; - Falta de motivación por parte de los compañeros de mayor rango, dificultades en el trabajo en equipo y falta de cooperación en casos de sospecha de maltrato; - Incertidumbre sobre la recepción de apoyo administrativo o jurídico por parte del hospital, carga de trabajo y limitaciones de tiempo.
Beck y Ogloff^(26)^	- Falta de pruebas suficientes; - Falta de confianza en los servicios de protección infantil.
Berchtold et al.^(28)^	- Dudas sobre la identificación, sensación de no ser responsable de la denuncia, incertidumbre sobre las consecuencias de la denuncia, protección parental, jerarquía médica y factores interprofesionales; - Falta de tiempo, olvido de la denuncia.
Egry et al.^(31)^	- Falta de conocimiento sobre los procesos de derivación y déficit de conocimiento sobre la violencia; - Necesidad de directrices de derivación más claras, red sanitaria insuficiente, falta de organización, recursos económicos y profesionales cualificados.
Elarousy y Abed^(32)^	- No saber cómo denunciar, creencia de poder intervenir de forma más eficaz que los equipos especializados; - Incertidumbre sobre el abuso, falta de corroboración por parte de otras personas, experiencias negativas tras la denuncia, percepción de que la lesión era leve y ausencia de consecuencias legales por no denunciar.
Gunn et al.^(37)^	- Desconocimiento de las leyes sobre denuncia de irregularidades; - Preocupación por las consecuencias para el cuidador o el niño, miedo a equivocarse, incertidumbre sobre la existencia del maltrato, malas experiencias previas con los servicios de protección infantil; - Tiempo y esfuerzo que supone la denuncia.
İnanici et al.^(38)^	- Desconocimiento de las normas de denuncia forense, dificultad para preparar el informe y desconocimiento de los procedimientos de notificación.
Kent et al.^(39)^	- Relaciones difíciles y cambios frecuentes de los trabajadores sociales, y falta de una respuesta adecuada tras las derivaciones.
Khanjari et al.^(40)^	- Restricciones éticas, sociales, culturales y religiosas, incluidos los conceptos de honor, modestia y vergüenza.
Leung et al.^(43)^	- Falta de pruebas suficientes, reticencia a recurrir a los sistemas legales, posible perjuicio para el niño o la familia derivado de la denuncia y preocupación por el anonimato; - El proceso de denuncia requiere mucho tiempo.
Lynne et al.^(47)^	- Desconocimiento de qué se debe denunciar o cómo hacerlo, e incertidumbre sobre el anonimato; - La creencia de que otras personas denunciarán el caso, el miedo a causar daño, experiencias negativas previas y la creencia en medidas drásticas por parte de los servicios sociales; - Falta de tiempo, retrasos en la denuncia y prioridad de las emergencias médicas.
Mandadi et al.^(48)^	- Conocimiento insuficiente de las leyes y los plazos de denuncia; - Tiempo insuficiente para la denuncia, falta de disponibilidad de trabajadores sociales, falta de respuesta o de actuación por parte de los servicios de protección infantil.
Mostovoy et al.^(49)^	- Falta de habilidades o formación; - Preocupación por los sentimientos del niño, creencia de que los niños no cooperarán, barreras culturales o lingüísticas, preocupaciones por la seguridad personal, creencia de que la notificación no servirá de nada y experiencias profesionales y personales negativas; - Falta de tiempo y deseo de evitar implicaciones legales.
Nouman et al.^(50)^	- Falta de pruebas de identificación y retroalimentación insuficiente por parte de los servicios de protección infantil.
Owaidah et al.^(9)^	- Incapacidad para identificar pruebas o indicadores, falta de conocimientos debido a una formación insuficiente, creencia de que las lesiones leves no justifican la denuncia y falta de confianza entre los profesionales sin experiencia; - Estigma, normas culturales, malentendidos sobre las leyes, falta de conciencia de las consecuencias, incertidumbre sobre la reacción de la familia,miedo a las consecuencias, experiencias negativas previas y miedo a causar más daño al niño; - Falta de una estructura normativa, implementación ineficaz de la formación y ausencia de implicaciones legales por no denunciar.
Patrick et al.^(53)^	- Preocupaciones por la seguridad del niño y la propia, incertidumbre en la identificación, confidencialidad y ramificaciones legales.
Regnaut et al.^(54)^	- Desconocimiento de los recursos profesionales; - Miedo a una identificación incorrecta, dificultad para controlar las emociones, sensación de soledad, miedo a las amenazas y factores socioeconómicos familiares; - Tiempo necesario para completar los informes, dificultad para prepararlos y obstáculos procedimentales para la denuncia de violaciones.
Riese et al.^(55)^	- Falta de formación; - Falta de tiempo y de recursos hospitalarios y extrahospitalarios.
Rowse^(56)^	- Falta de respuesta o de medidas positivas por parte de los trabajadores sociales.
Roy et al.^(57)^	- Falta de tiempo; - Preocupaciones por la confidencialidad.
Russell et al.^(58)^	- Incertidumbre a la hora de informar; - Miedo a una identificación incorrecta, a la burocracia, a la jerarquía, a la falta de sensibilidad y al apoyo de los servicios sociales y los compañeros; - Falta de directrices, carga de trabajo y largos procedimientos de notificación.
Tiyyagura et al.^(61)^	- Miedo a cometer errores y a las reacciones de los cuidadores; - Entorno de trabajo con un ritmo acelerado.
Vilensky et al.^(63)^	- Falta de conocimientos, formación y conocimientos jurídicos; - Incertidumbre a la hora de identificar y notificar las consecuencias, miedo a las repercusiones para el paciente, la familia o el personal, y miedo a una notificación errónea.
Wißmann et al.^(64)^	- Falta de experiencia en la identificación o el manejo de casos; - Miedo a las acusaciones falsas, incertidumbre en la identificación, creencia en un hecho aislado, reticencia debido a los vínculos personales con los padres, miedo a testificar ante la justicia, preocupaciones por la seguridad, explicaciones culturales o religiosas sobre las lesiones y desconfianza en la eficacia de los servicios de protección infantil; - La falta de tiempo, la insuficiencia de las intervenciones de los servicios de protección infantil, las barreras administrativas y la creencia de que el trabajo propio es más eficaz.
Willis y Horner^(65)^	- Desconfianza en la prevalencia del maltrato y temor a las consecuencias negativas de denunciarlo; - Falta de confianza en la capacidad de los servicios sociales para gestionar los casos.
Wilson y Lee^(19)^	- Desconocimiento sobre cómo denunciar los casos, déficit en habilidades clínicas y de juicio, y falta de concienciación; - Normas culturales, proximidad rural que reduce el anonimato, opiniones profesionales sesgadas, reticencia a denunciar, desconfianza en las agencias, miedo a las disputas y dificultades emocionales; - Infraestructura de denuncia deficiente, falta de un sistema centralizado, mala comunicación, mantenimiento de registros inadecuado, falta de retroalimentación, ausencia de directrices explícitas, herramientas de evaluación inadecuadas, apoyo de la dirección insuficiente, alta rotación de personal, carga de trabajo y falta de protección para los denunciantes.

**Cuadro 6:** Barreras para intervenir en casos de violencia contra los niños – Lisboa, Portugal, 2025.

Estudio	Barreras para intervenir en casos de violencia contra los niños
Beck et al.^(27)^	- Falta de formación sobre el tráfico sexual y falta de concienciación sobre el fenómeno; - Delicadeza del tema y miedo de las víctimas a denunciar; - Falta de políticas o directrices organizativas y falta de financiación o recursos.
Egry et al.^(31)^	- Rendimiento de la red obstaculizado por la lógica sectorial y vertical, y por una comunicación y un intercambio de información lentos y desequilibrados.
Faryan et al.^(34)^	- Falta de conocimientos por parte de los profesionales; - Factores culturales; - Falta de recursos y capacidad organizativa, dificultades de implementación y condiciones de trabajo inadecuadas del personal de primera línea.
Kent et al.^(39)^	- Preocupación por que la participación en el seguimiento y la vigilancia de las familias identificadas como «en riesgo» pudiera suponer una amenaza para la imagen amable de las enfermeras de salud pública.
Land y Barclay^(42)^	- Toma de decisiones subjetiva y falta de formación adecuada; - Perspectivas culturales, protección profesional, seguridad personal e implicaciones profesionales; - Escasez de recursos, falta de colaboración multidisciplinar, falta de protocolos claros, falta de autonomía y estructuras jerárquicas.
Lines et al.^(44)^	- Miedo a cometer errores y sistemas inflexibles; - Mala interconexión de los servicios, duplicación, mala coordinación, sistemas rígidos y jerarquías en el intercambio de información.
Rowse^(56)^	- Brecha entre la teoría y la práctica, falta de conocimientos y de apoyo en los procedimientos formales; - Falta de información sobre la evolución de los casos.
Solem et al.^(18)^	- Dificultad para definir e identificar la negligencia, formación insuficiente y falta de uso de herramientas debido a la elevada rotación del personal; - Recursos limitados, elevada rotación del personal, número excesivo de casos que afecta a la gestión de los planes de protección infantil y falta de supervisión; - No se escuchan ni se tienen en cuenta las opiniones de los niños; los profesionales sucumben a la «regla del optimismo».
Tiyyagura et al.^(62)^	- Variabilidad del apoyo institucional a los defensores, dependencia de la motivación individual, sobrecarga de funciones de los defensores, rotación de los defensores y variabilidad de los conocimientos sobre el programa por parte de los proveedores de primera línea.

Los estudios incluidos en esta revisión se publicaron entre 1987 y 2024, teniendo en cuenta el periodo de búsqueda realizado entre julio y octubre de 2024, en 20 países diferentes: Estados Unidos de América (13)^(19,27,29,33,35,37,47,48,55,60,61,62,65)^; Arabia Saudita (3)^(9,32,34)^; Australia (4)^(42,44,45,58)^; Brasil (3)^(31,36,46)^; Israel (3)^(49,50,63)^; Ghana (2)^(24,25)^; Finlandia (2)^(51,52)^; Inglaterra (2)^(56,59)^; Reino Unido (2)^(53,57)^; Canadá (1)^(26)^; Alemania (1)^(64)^; Colombia (1)^(30)^; Francia (1)^(54)^; Países Bajos (1)^(41)^; China (1)^(43)^; Irlanda del Norte (1)^(58)^; Irlanda (1)^(39)^; Irán (1)^(40)^; Suiza (1)^(28)^; y Turquía (1)^(38)^. Esto se traduce en una considerable diversidad sociocultural que, a su vez, nos permite adoptar una visión más integral y completa.

Los estudios incluidos se llevaron a cabo en 20 países, abarcando diferentes continentes y contextos socioculturales. Más concretamente, los estudios procedían de América del Norte (p. ej., Estados Unidos, Canadá), Europa (p. ej., Reino Unido, Finlandia, Países Bajos, Alemania, Francia, Suiza, Irlanda), Asia (p. ej., Arabia Saudita, Irán, China), Oriente Medio (p. ej., Israel), África (p. ej., Ghana) y América del Sur (p. ej., Brasil). La distribución temporal de los estudios abarcó desde 1987 hasta 2024, con una mayor concentración de estudios publicados en la última década, lo que refleja una mayor atención a la protección infantil y a las responsabilidades profesionales en los entornos de atención médica. Esta diversidad geográfica y temporal pone de relieve la amplitud de los contextos en los que se han identificado barreras para la prevención de la violencia contra los niños.

En cuanto al diseño de los estudios, se identificaron 18 estudios cualitativos^(24,25,30,31,34,36,39,41,42,44,45,46,50,56,60,61,62,63)^, incluyendo un estudio de caso^(46)^ y un estudio fenomenológico^(34)^. También se incluyeron un proyecto de mejora de la calidad^(29)^, un análisis de respuestas cualitativas^(45)^ y una auditoría^(53)^. Se incluyó un estudio descriptivo^(32)^. Además, se incluyeron un estudio cuantitativo^(52)^, una encuesta correlacional cuantitativa^(49)^, dos estudios transversales^(43,48)^, un estudio epidemiológico^(55)^ y un estudio cuasi- experimental^(40)^. Por último, 15 estudios emplearon enfoques de métodos mixtos^(26,27,28,33,35,37,38,47,51,54,59,64,65)^. Tres de los estudios incluidos son revisiones de la literatura^(9,18,19)^.

En cuanto al lugar de recopilación de datos, en 20 estudios los datos se recopilaron en entornos hospitalarios^(24,25,26,28,29,30,32,40,44,47,48,51,52,53,55,56,57,59,60,62)^. De estos, dos se refieren a hospitales universitarios^(51,52)^; uno incluye datos recopilados en tres hospitales comunitarios y un centro médico académico^(62)^; y uno se refiere a un gran hospital universitario metropolitano, un pequeño hospital metropolitano y un hospital rural^(59)^. Dos estudios se realizaron en entornos de urgencias generales^(47,60)^, cuatro en hospitales pediátricos^(30,31,32,55,56)^, tres en servicios de urgencias pediátricas^(28,29,48)^ y uno tanto en un servicio de urgencias pediátricas como en una sala de pediatría^(40)^. Además, dos estudios recopilaron datos en clínicas privadas^(54,64)^ y uno en un entorno prehospitalario^(61)^. Un estudio no especificó el servicio de salud donde se recopilaron los datos^(43)^, y otro solo informó que los datos se recopilaron de una organización de salud infantil^(41)^.

En ocho estudios, los datos se recopilaron en entornos de atención de salud comunitaria^(31,36,38,39,42,46,58,63)^, y en dos estudios, los datos se obtuvieron tanto de entornos comunitarios como hospitalarios^(44,45)^. En nueve estudios, los datos se recopilaron de personas que trabajan con niños o que están afiliadas a organizaciones de atención médica o de protección infantil^(26,33,34,35,37,45,49,50,65)^.

En cuanto al tipo de violencia contra los niños, seis estudios tratan el maltrato infantil en general^(28,41,43,49,54,63)^; un artículo aborda el abandono^(18)^; y 14 profundizan en el maltrato y el abandono infantil^(9,19,32,37,38,42,44,48,50,53,60,61,64)^. Cinco de los estudios abarcan el maltrato físico^(24,25,29,30,58)^, y uno examina el maltrato físico y emocional^(35)^. En cuanto al abuso sexual contra los niños, un artículo examina el abuso sexual infantil^(65)^, y otro explora el tráfico sexual infantil^(26)^. Cinco estudios abordan tres o más tipos de violencia contra los niños, todos los cuales incluyen el maltrato físico y el abandono, pero también el maltrato emocional y psicológico^(26,51)^, el abuso sexual^(26,36,51,59)^ y el abandono^(46)^. Uno examina la violencia doméstica y el maltrato^(57)^, y otro la violencia juvenil^(55)^. Nueve de los artículos incluidos no especifican el tipo de abuso^(31,33,34,39,40,45,47,52,56)^.

### Barreras Para la Prevención de la Violencia Contra los Niños

Esta revisión identificó cuatro tipos principales de barreras relacionadas con la violencia contra los niños ((i) barreras para la prevención; (ii) barreras para la identificación; (iii) barreras para la denuncia; y (iv) barreras para la intervención), tal como se detalla en las Cuadros 3, 4, 5 y 6, y se sintetiza visualmente en la Figura 2.

**Figura 2:** Mapa conceptual de las barreras identificadas por los profesionales de la salud en la prevención de la violencia contra los niños – Lisboa, Portugal, 2025.

Para mejorar la claridad analítica, los cuatro tipos de barreras (prevención, identificación, notificación e intervención) se sintetizaron aún más en tres dimensiones transversales —deficiencias en el desempeño profesional, barreras contextuales y limitaciones organizativas y de recursos—, tal y como se ilustra en la Figura 2.

## DISCUSIÓN

Esta revisión de alcance identificó cuatro tipos principales de barreras relacionadas con la violencia contra los niños — prevención, identificación, denuncia e intervención—, que reflejan diferentes etapas de la acción profesional en materia de protección infantil. Más allá de esta organización por categorías, la síntesis de los hallazgos reveló que estas barreras se agrupaban en torno a tres dimensiones transversales: deficiencias en el desempeño profesional; barreras contextuales; y limitaciones organizativas y de recursos. Esta estructura analítica, ilustrada en la Figura 2, permite una comprensión más integrada de cómo operan las barreras a lo largo de las diferentes fases de la prevención y la respuesta a la violencia contra los niños.

La inclusión de estudios realizados en 20 países de varios continentes subraya la considerable heterogeneidad sociocultural, jurídica y organizativa que subyace a estas barreras. Aunque la manifestación específica de las barreras varía según los contextos regionales y las estructuras de los sistemas de salud, surgieron patrones similares, lo que sugiere que muchos de los desafíos a los que se enfrentan los profesionales de la salud pueden ser transversales. En particular, las deficiencias en el desempeño profesional y las limitaciones organizativas se señalaron con mayor frecuencia que las barreras contextuales, lo que indica su peso relativo a la hora de limitar las prácticas efectivas de prevención, identificación, denuncia e intervención.

La distribución desigual de los estudios entre los distintos tipos de barreras —siendo las barreras de denuncia e identificación las más frecuentemente señaladas— puede reflejar una mayor complejidad institucional, jurídica y emocional asociada a estas etapas de la protección infantil. Este hallazgo es particularmente relevante cuando se interpreta a la luz del Objetivo de Desarrollo Sostenible 16.2, que hace hincapié en la necesidad de fortalecer la capacidad institucional, la capacitación profesional y la coordinación intersectorial para eliminar todas las formas de violencia contra los niños. La persistencia de barreras similares en diversos contextos y a lo largo del tiempo sugiere que el progreso hacia este objetivo sigue siendo desigual y que los desafíos sistémicos parecen continuar limitando la acción profesional.

En lugar de abordar las barreras exclusivamente según las etapas de la intervención, el análisis que sigue sintetiza los hallazgos en torno a tres dimensiones transversales —brechas en el desempeño profesional, barreras contextuales y limitaciones organizativas y de recursos— con el fin de ampliar el debate y resaltar sus interrelaciones.

### Barreras Para la Prevención de la Violencia Contra los Niños

Se identifican barreras para la prevención de la violencia contra los niños en cinco de los estudios incluidos^(31,35,39,41,45)^. Tres estudios identificaron brechas relacionadas con el desempeño profesional como una barrera para prevenir la violencia contra los niños^(35,41,45)^. Estas incluyen: falta de capacitación, conocimiento y confianza para asesorar a los padres sobre la disciplina infantil^(35)^; escasa familiaridad con las directrices y baja autoeficacia en situaciones que implican signos ambiguos, comunicación con los cuidadores, planificación del seguimiento e intercambio de información entre agencias^(41)^; y la necesidad de desarrollo profesional con acceso a supervisión clínica en entornos de aprendizaje que brinden apoyo^(45)^.

Se informaron barreras contextuales en tres estudios^(35,41,45)^, entre ellas: preocupaciones sobre la sensibilidad cultural, la percepción de que el tema tiene baja prioridad, la incomodidad al abordarlo y las dudas sobre la eficacia de la intervención^(35)^; la falta de voluntad o la incapacidad de los cuidadores para cooperar y una mayor incertidumbre al discutir la sospecha de abuso en el entorno doméstico^(41)^; e influencias psicosociales como el agotamiento, la fatiga por compasión y factores personales, la exclusión de las discusiones sobre protección y un desajuste entre las prácticas estandarizadas y las necesidades de la familia^(45)^.

Cuatro estudios describieron limitaciones organizativas y de recursos^(31,35,41,45)^. Entre ellas se incluyen: el número reducido de profesionales^(31)^; la disponibilidad de tiempo limitada, la ausencia de reembolso financiero y el apoyo institucional insuficiente^(35)^; deficiencias en los registros médicos electrónicos y una cooperación interinstitucional deficiente^(41)^; y cargas de trabajo elevadas, expectativas poco realistas, falta de personal, brechas estructurales y de comunicación, ausencia de acuerdos interdepartamentales y disponibilidad limitada de servicios^(45)^.

### Barreras Para Identificar la Violencia Contra los Niños

Veintiún estudios incluidos en esta revisión identifican barreras para identificar la violencia contra los niños^(25,27,28,29,30,31,33,36,38,40,46,48,51,52,54,55,58,59,60,61,65)^.

Dieciocho estudios identificaron las deficiencias en el desempeño profesional como barreras para identificar la violencia contra los niños^(27,28,29,30,31,33,36,38,40,48,51,52,54,55,58,59,60,61)^. Entre ellas se incluyen: falta de capacitación y sensibilización^(27,55,59)^; conocimiento, experiencia y confianza insuficientes para detectar signos y síntomas de maltrato^(28,33,36,40,48,52,58)^; dificultad para distinguir entre lesiones accidentales e intencionales^(28,61)^; escaso conocimiento de las directrices y herramientas^(29,48)^; incapacidad para reconocer indicadores conductuales de negligencia^(60)^; habilidades de comunicación inadecuadas con los niños y los cuidadores^(30,33,38)^; técnicas de entrevista deficientes^(58)^; incertidumbre en las presentaciones clínicas^(48)^; e incomodidad con los casos pediátricos^(61)^.

Catorce estudios informaron sobre barreras contextuales^(25,27,29,30,33,36,38,46,51,52,58,60,65)^. Entre ellas se incluyen: la naturaleza sensible o tabú del tema^(27,28,52)^; el encubrimiento o la negación por parte de las familias^(36,46,52)^; factores socioeconómicos que enmascaran el abandono^(46)^; la difusión de la responsabilidad^(25)^; la incertidumbre en la interpretación de las pruebas de detección^(29)^; la percepción de que las herramientas son innecesarias^(30)^; barreras lingüísticas y culturales^(33)^; la renuencia de los profesionales a creer que se produce el abuso^(52,65)^; el deseo de creer a los cuidadores^(60)^; los prejuicios personales^(60)^; el temor a las consecuencias de la denuncia^(65)^; el temor a una identificación incorrecta^(38,58)^; el temor al daño^(38)^; y los desafíos de los entornos de emergencia, como la falta de contacto continuo con la familia^(60)^.

Dieciséis estudios describieron limitaciones organizativas y de recursos^(27,28,29,30,31,33,36,38,48,51,52,55,58,59,61,65)^. Entre ellas se incluyen: la falta de políticas o directrices organizativas^(27,51)^; recursos limitados o inexistentes^(27,55,59)^; personal o tiempo insuficientes^(29,30,55,59)^; coordinación interdepartamental o interinstitucional deficiente^(29,48)^; retrasos en las consultas^(29)^; infraestructura de documentación inadecuada^(29)^; ausencia de herramientas validadas^(48)^; falta de protocolos de identificación^(33)^; necesidad de fortalecer la atención primaria de salud como punto de referencia^(31)^; cargas de trabajo pesadas^(36,38,59)^; y falta de confianza en la capacidad de los servicios sociales para gestionar los casos^(65)^.

### Barreras Para la Denuncia de la Violencia Contra los Niños

Veintisiete estudios incluidos en esta revisión identifican barreras para la denuncia de la violencia contra los niños^(9,19,24,25,26,28,31,32,37,38,39,40,43,47,48,49,50,53,54,55,56,57,58,61,63,64,65)^.

Se identificaron deficiencias en el desempeño profesional en 21 estudios^(9,19,25,26,28,31,32,37,38,40,47,48,49, 53,54,55,56,58,63,64,65)^. Entre los problemas señalados se incluyen el conocimiento o las habilidades insuficientes en los procedimientos de identificación y notificación^(9,19,25,31,32,37,38,47,48,49,54,55,58,63,64)^, la dificultad para preparar informes^(38,54)^ y la falta de confianza o experiencia^(9,28,64)^. Otras barreras incluyen la toma de decisiones subjetiva^(58)^, la incertidumbre sobre qué se debe notificar^(47,58)^ y la creencia de que se puede intervenir de manera más eficaz sin una notificación formal^(32)^.

Se observaron barreras contextuales en veinticinco estudios^(9,19,24,25,26,28,29,31,32,37,40,43,46,47,48,49,53,54,55,56,57,58,61,63,64,65)^. Entre ellas se incluyen: el temor a sufrir daño físico o espiritual^(9,24,53,54,64)^; el temor a una identificación incorrecta^(54,58,63)^; el estigma y las normas culturales o religiosas^(19,9,40,64)^; la falta de pruebas que lo corroboren^(26,32,43)^; y la preocupación por las consecuencias negativas que la denuncia pueda tener para el profesional, el niño o la familia^(37,43,47,49,58,61,63,65)^. Otras barreras incluyen la desconfianza en los servicios de protección infantil^(64,65)^, la renuencia a interactuar con los sistemas legales^(43,49)^, la creencia de que otros denunciarán^(47)^, los desafíos emocionales^(19)^ y las dinámicas jerárquicas o interprofesionales que desalientan la denuncia^(28,58)^.

En 23 estudios se informaron limitaciones organizativas y de recursos^(9,19,25,26,28,29,31,37,39,43,46,47,48,49,50,54,55,56,57,58,61,64,65)^. Entre las barreras señaladas se incluyen: la falta de políticas organizativas, directrices claras o protocolos estructurados^(19,26,31,58)^; la falta de tiempo y las elevadas cargas de trabajo^(9,25,28,37,43,47,48,49,54,55,57,58,61,64)^; la insuficiencia de recursos o de personal^(19,31,48,50)^; comunicación o retroalimentación interinstitucional deficiente^(19,39,50,56)^; y barreras procedimentales o administrativas^(54,58,64)^. Otras limitaciones incluyen la implementación ineficaz de la capacitación^(9)^, la ausencia de consecuencias legales por no denunciar^(9)^ y la falta de protección para quienes denuncian^(19)^.

### Barreras Para Intervenir en Casos de Violencia Contra los Niños

Nueve estudios incluidos en esta revisión identifican barreras para intervenir en casos de violencia contra los niños^(18,27,31,34,39,42,44,56,62)^.

Se informaron deficiencias en el desempeño profesional en seis estudios^(18,27,34,42,56,62)^. Entre ellas se incluyen: la falta de capacitación y de conciencia sobre formas específicas de violencia, como el tráfico sexual^(27)^; el conocimiento insuficiente en la práctica profesional general^(34)^; la toma de decisiones subjetiva y la falta de capacitación adecuada^(42)^; las brechas entre el conocimiento teórico y la aplicación práctica, así como la falta de apoyo en los procedimientos formales^(56)^; la dificultad para definir e identificar la negligencia; la capacitación insuficiente; y la incapacidad para utilizar los conjuntos de herramientas debido a la alta rotación de personal^(18)^.

Se identificaron barreras contextuales en seis estudios^(18,27,34,39,42,44)^. Entre ellas se incluyen: la sensibilidad del tema y el miedo de las víctimas a denunciar^(27)^; factores culturales que influyen en la actuación profesional^(34,42)^; la preocupación de que el seguimiento de las familias «en riesgo» pudiera dañar la imagen amigable de la enfermera^(39)^; el miedo a cometer errores y a lidiar con sistemas inflexibles^(44)^; y situaciones en las que no se tienen en cuenta las opiniones de los niños o en las que los profesionales se adhieren a una «regla del optimismo»^(18)^.

Las limitaciones organizativas y de recursos se mencionaron en ocho estudios^(18,27,31,34,42,44,56,62)^. Entre ellas se incluyen: la falta de políticas y orientación organizativas, y la financiación o los recursos insuficientes^(27)^; el bajo rendimiento de la red debido a estructuras sectoriales y verticales y a una comunicación desequilibrada^(31)^; la falta de recursos y capacidad organizativa, los desafíos de implementación y las condiciones de trabajo inadecuadas para el personal de primera línea^(34)^; los recursos escasos, la ausencia de colaboración multidisciplinaria, la falta de protocolos claros, la autonomía limitada y las estructuras jerárquicas^(42)^; la mala interconexión de los servicios, la duplicación, la mala coordinación, los sistemas rígidos y las jerarquías en el intercambio de información^(44)^; la falta de información sobre la evolución de los casos^(56)^; los recursos limitados, la alta rotación de personal, el exceso de casos que afecta a la gestión de los planes de protección infantil y la falta de supervisión^(18)^; y la variabilidad en el apoyo institucional a los defensores, la dependencia de la motivación individual, la sobrecarga de funciones, la rotación de los defensores y el conocimiento inconsistente del programa entre los proveedores de primera línea^(62)^.

## CONCLUSIÓN

Esta revisión de alcance identificó múltiples barreras interconectadas a las que se enfrentan los profesionales de la salud en la prevención, identificación, denuncia e intervención en casos de violencia contra los niños. Estas barreras se clasifican en tres dimensiones principales: deficiencias en el desempeño profesional; desafíos contextuales; y limitaciones organizativas y relacionadas con los recursos. Las limitaciones identificadas incluyen déficits en el conocimiento y la capacitación, incertidumbre sobre los procedimientos legales, la influencia de las normas culturales, la ausencia de protocolos claros y el apoyo institucional insuficiente.

A pesar de la amplitud de la evidencia analizada, esta revisión presenta limitaciones metodológicas, entre ellas la ausencia de una evaluación de la calidad de los estudios incluidos y la heterogeneidad metodológica, lo que puede haber limitado la identificación de matices relevantes. Sin embargo, de acuerdo con las recomendaciones del JBI para las revisiones exploratorias, la evaluación crítica de la calidad metodológica no es obligatoria, ya que el objetivo principal es mapear el alcance y la naturaleza de la evidencia disponible, más que evaluar la eficacia de las intervenciones. Además, dado que las búsquedas se realizaron dentro de un intervalo de tiempo definido y no se actualizaron continuamente, es posible que no se hayan captado estudios relevantes publicados después del período de búsqueda. Aunque se implementó una estrategia de búsqueda exhaustiva en múltiples bases de datos, no se puede descartar la posibilidad de que se hayan omitido estudios elegibles. Por otra parte, aunque los criterios de elegibilidad abarcaban una amplia gama de profesionales de la salud y afines involucrados en la protección infantil, la estrategia de búsqueda empleó principalmente términos relacionados con el personal de salud y la enfermería pediátrica. Esto puede haber limitado la recuperación de estudios en los que participaran profesionales de otros campos, como las ciencias sociales o humanas, lo que podría haber restringido la amplitud de las perspectivas profesionales captadas. Las variaciones en el diseño de los estudios, los grupos profesionales y los entornos contextuales también introdujeron una heterogeneidad sustancial, lo que puede haber influido en la síntesis de los resultados. Estos factores deben tenerse en cuenta al interpretar los hallazgos de esta revisión de alcance.

No obstante, a pesar de las diferencias metodológicas y contextuales, surgieron varias barreras de manera consistente en toda la literatura, lo que sugiere que ciertos desafíos estructurales y profesionales son generalizados y pueden constituir obstáculos globales para la prevención, identificación, denuncia e intervención efectivas en casos de violencia contra los niños. Esta diversidad enriquece la comprensión de cómo se manifiestan dichas barreras en diferentes sistemas de salud y contextos socioculturales, lo que respalda el desarrollo de estrategias sensibles al contexto para el desarrollo de capacidades profesionales y la formulación de políticas.

Se recomienda invertir en formación continua y específica, mejorar las condiciones de trabajo y reforzar la colaboración interinstitucional. Las investigaciones futuras deberían centrarse en el desarrollo y la evaluación de intervenciones que mejoren la capacidad de respuesta de los profesionales, en particular mediante la evaluación del impacto de los programas de capacitación y las estrategias de colaboración adaptadas a diferentes contextos culturales y geográficos. Además, se debería prestar mayor atención a las intervenciones basadas en la comunidad y a los diseños de investigación longitudinales, que pueden ayudar a captar cómo evolucionan con el tiempo las barreras para la prevención de la violencia contra los niños, así como a evaluar la sostenibilidad y la eficacia a largo plazo de las intervenciones en diferentes entornos.

## Data Availability

El conjunto de datos que respaldan las conclusiones de este estudio está disponible previa solicitud al autor correspondiente.
